# Loss of direct adrenergic innervation after peripheral nerve injury causes lymph node expansion through IFN-γ

**DOI:** 10.1084/jem.20202377

**Published:** 2021-06-04

**Authors:** Chien-Sin Chen, Jasmin Weber, Stephan Jonas Holtkamp, Louise Madeleine Ince, Alba de Juan, Chen Wang, Lydia Lutes, Coline Barnoud, Burak Kizil, Sophia Martina Hergenhan, Johanna Salvermoser, Manuel Lasch, Elisabeth Deindl, Barbara Schraml, Dirk Baumjohann, Christoph Scheiermann

**Affiliations:** 1 Biomedical Center, Institute of Cardiovascular Physiology and Pathophysiology, Faculty of Medicine, Ludwig-Maximillians-Universität München, Planegg-Martinsried, Germany; 2 Department of Pathology and Immunology, Faculty of Medicine, University of Geneva, Geneva, Switzerland; 3 Walter-Brendel-Centre of Experimental Medicine, University Hospital, Ludwig-Maximillians-Universität München, Munich, Germany; 4 Department of Otorhinolaryngology, Head and Neck Surgery, University Hospital, Ludwig-Maximillians-Universität München, Munich, Germany; 5 Institute for Immunology, Biomedical Center, Faculty of Medicine, Ludwig-Maximillians-Universität München, Planegg-Martinsried, Germany; 6 Medical Clinic III for Oncology, Hematology, Immuno-Oncology and Rheumatology, University Hospital Bonn, University of Bonn, Bonn, Germany

## Abstract

Peripheral nerve injury can cause debilitating disease and immune cell–mediated destruction of the affected nerve. While the focus has been on the nerve-regenerative response, the effect of loss of innervation on lymph node function is unclear. Here, we show that the popliteal lymph node (popLN) receives direct neural input from the sciatic nerve and that sciatic denervation causes lymph node expansion. Loss of sympathetic, adrenergic tone induces the expression of IFN-γ in LN CD8 T cells, which is responsible for LN expansion. Surgery-induced IFN-γ expression and expansion can be rescued by β2 adrenergic receptor agonists but not sensory nerve agonists. These data demonstrate the mechanisms governing the pro-inflammatory effect of loss of direct adrenergic input on lymph node function.

## Teaser

This study shows loss of sympathetic innervation to the popliteal lymph node to induce IFN-γ expression in CD8 T cells, causing expansion. This is rescued by β2 adrenergic receptor agonists, demonstrating the pro-inflammatory effect of loss of direct adrenergic input.

## Introduction

Peripheral nerve injury has primarily been linked to loss of motor functions and impaired muscle activity (Hutson and Di Giovanni, 2019; Mahar and Cavalli, 2018). At the injury site, changes in nerve structure induce the infiltration of innate immune cells, causing an inflammatory response that degrades the myelin sheath, ultimately allowing regrowth of the nerve (Rotshenker, 2011). While most studies have focused on the immune response within the nerve in order to better understand the mechanisms involved and design therapies for the restoration of motor function, the effect of loss of neural tone on LN function has largely been ignored.

However, assessing the effects of acute and chronic denervation on the immune system, and specifically the LN as one of its pivotal organs, is critical, as patients with nerve injuries often exhibit local or systemic immunosuppression. This has been attributed to an intricate interplay between the nervous and the immune system and loss of neural substances, which can directly or indirectly affect the immune system (Kipnis and Filiano, 2018; Elenkov et al., 2000). Spinal cord injury as well as stroke can lead to microbial infections (DeVivo et al., 1989; Soden et al., 2000; Langhorne et al., 2000), which has been linked to defective autonomic nerve signaling (Meisel et al., 2005; Prass et al., 2003; Woiciechowsky et al., 1998), and is the leading cause of death in these patients. In a mouse model, experimental stroke has been shown to trigger the release of the sympathetic neurotransmitter norepinephrine, causing invariant natural killer (NK) T cells within liver sinusoids to downmodulate inflammatory cytokine production, whose function is to prevent microbial infections (Wong et al., 2011). The sympathetic nervous system (SNS) has thus emerged as a key component in the modulation of the immune system, both in steady-state and inflammation (Elenkov et al., 2000).

LNs are richly innervated tissues (van de Pavert et al., 2009), receiving both sympathetic and sensory nerve input (Felten et al., 1987; Felten et al., 1985; Felten et al., 1984; Huang et al., 2021). However, the role of local neural tone and specifically the contribution of sympathetic versus sensory nervous input for LN function remain largely unknown. Recent data indicate that activation of sensory nerve subsets can cause different effects in the LN (Huang et al., 2021). In contrast, lack of input as is the case after injury has not been studied. Specifically, a detailed analysis of the effects of denervation on LN function is lacking. Stimulated by the evidence of LN innervation, we postulated that direct neural tone modulates the immune response in LNs. Here, we defined the mechanistic chain of events in the LN response caused by lack of direct neural input after peripheral nerve injury.

## Results

### Sciatic denervation causes LN expansion

**Figure 1.**
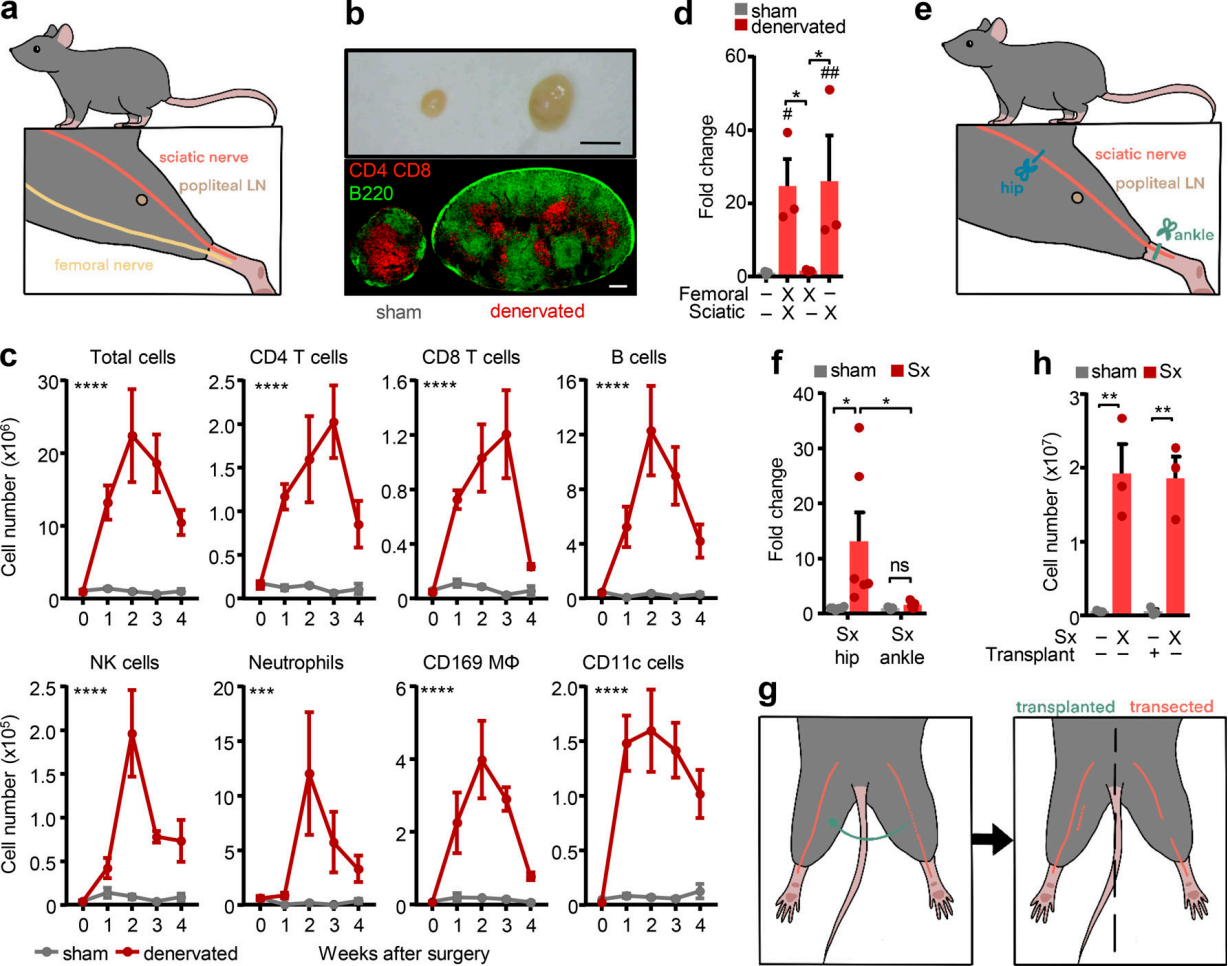
**Sciatic denervation causes LN expansion. (a)** Overview of the femoral and sciatic surgical denervation strategies and location of the popLN in the murine leg. **(b)** Macroscopic view (top) and T (red) and B (green) cell staining (bottom) of sham-operated and denervated popLNs, 2 wk after unilateral surgical denervation. Scale bars, 2 mm (top) and 200 µm (bottom). **(c)** Time course (in weeks) of leukocyte subsets in sham-operated and denervated popLNs after unilateral surgical denervation; *n* = 3–8 mice from two independent experiments, two-way ANOVA. Asterisks indicate significance between sham-operated and denervated popLNs. **(d)** Fold change of cellularity in the popLN in the indicated unilateral denervation settings compared with the sham-operated sides; *n* = 3–9 mice; data are representative of two independent experiments; one-way ANOVA; Tukey’s post-test. # represents statistical differences between indicated groups and the sham group. **(e)** Overview of the sciatic surgical denervation strategies at hip or ankle level. **(f)** Fold change of cellularity in the popLN after unilateral sciatic denervation (Sx) at hip or ankle level compared with its sham-operated counterpart; *n* = 5 or 6 mice from two independent experiments; two-way ANOVA; Šídák’s post-test. **(g)** Overview of the sciatic surgical denervation strategy in combination with autologous transplantation of a sciatic nerve segment to the contra-lateral side. **(h)** Cellularity of the popLN after unilateral sciatic denervation with or without transplantation of a sciatic nerve segment to the contralateral side; *n* = 3 mice; data are representative of two independent experiments; two-way ANOVA; Šídák’s post-test. All mice in this figure were 8–12-wk-old WT males purchased from Charles River or Janvier Labs. All data are presented as mean ± SEM; *, #, P < 0.05; **, ##, P < 0.01; ***, P < 0.001; ****, P < 0.0001. Mϕ, macrophage. # represents statistical differences between indicated groups and the sham group.

**Figure S1.**
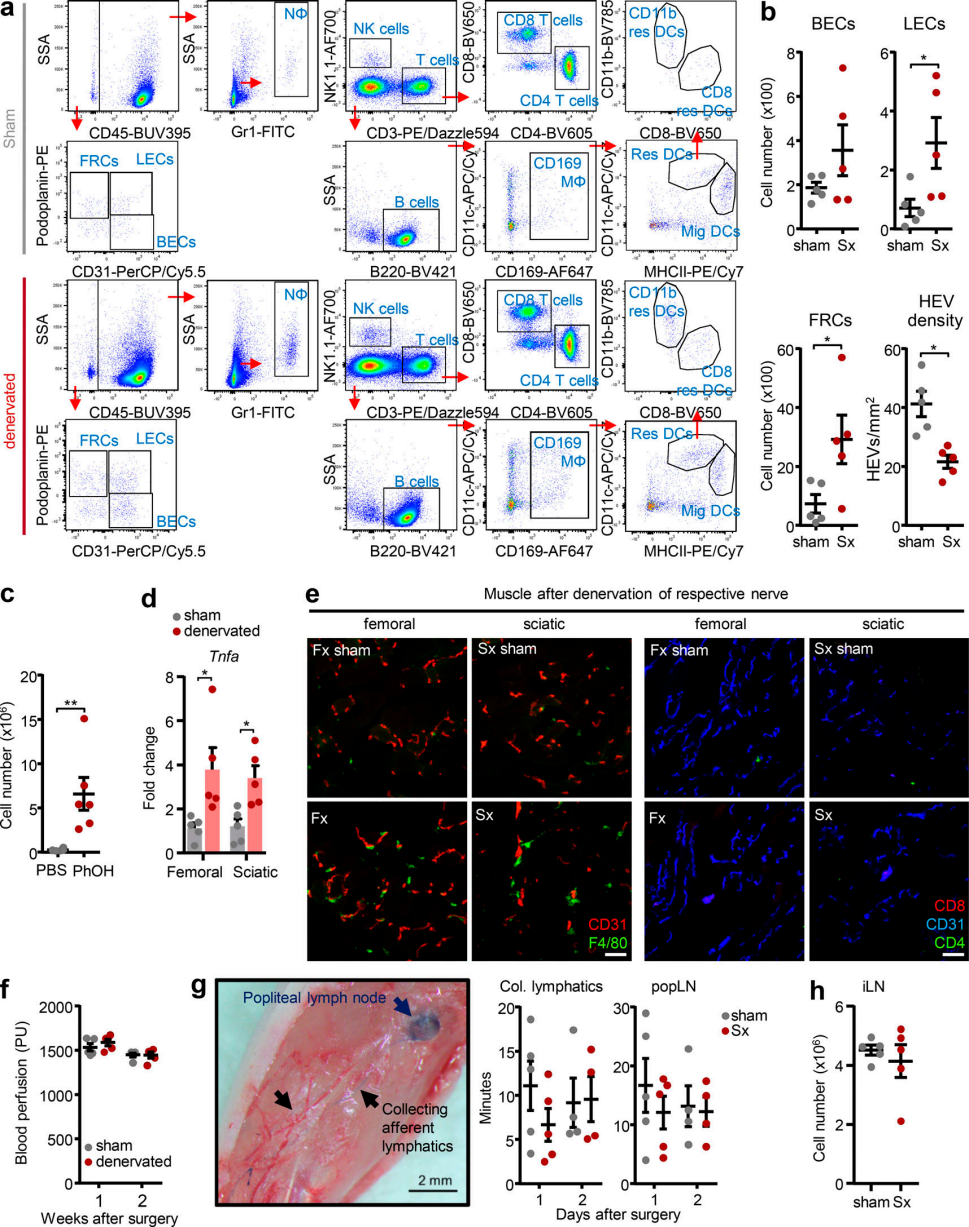
**Overview of gating strategies and effects on circulating systems and the stromal compartment. (a)** Gating strategy for flow cytometry on sham-operated and denervated popLN after debris and doublet exclusions and live/dead staining. **(b)** Cellularity of stromal cells (BEC, blood endothelial cells; LEC, lymphatic endothelial cells; FRC, fibroblastic reticular cells) and quantification of HEV density in the popLN 1 wk after unilateral sciatic denervation; *n* = 5 mice; data are representative of two independent experiments; unpaired Student’s *t* test. **(c)** Cellularity of the popLN 1 wk after phenol-mediated neurolysis of the sciatic nerve; *n* = 6 mice from two independent experiments; unpaired Student’s *t* test. **(d)** Expression of *Tnfa* in the muscle surrounding the popLN with or without surgical denervation of the indicated nerves; *n* = 5 mice; data are representative of two independent experiments; two-way ANOVA; Šídák’s post-test. **(e)** Presence of F4/80^+^ macrophages but not T cells in the muscle surrounding the popLN with or without surgical denervation of the indicated nerves. **(f)** Blood perfusion analysis in the paw 1 and 2 wk after unilateral sciatic denervation; *n* = 5 mice; data are representative of two independent experiments; unpaired Student’s *t* test. PU, perfusion units. **(g)** Image (left) and quantification (right) of accumulation of Evans Blue dye in collecting afferent lymphatic vessels and the popLN 1 or 2 d after unilateral sciatic denervation; *n* = 4 or 5 mice from two independent experiments; two-way ANOVA; Šídák’s post-test. **(h)** Cellularity of the inguinal LN (iLN) 1 wk after sham surgery or unilateral sciatic denervation; *n* = 5 mice; data are representative of two independent experiments. All mice in this figure were 8–12-wk-old WT males purchased from Charles River or Janvier Labs. All data are presented as mean ± SEM; *, P < 0.05; **, P < 0.01. Col., collecting; Fx, femoral denervation; Mϕ, macrophage; Mig, migratory DC; Nϕ, neutrophil; Res, resident DC; SSA, side scatter area; Sx, sciatic denervation.

As a model of peripheral nerve injury, we investigated the role of neural innervation on LN function by surgically transecting the sciatic and femoral nerves unilaterally at hip level while performing a sham surgery on the contra-lateral side (Fig. 1 a). The effect of the surgery was quantified in the popliteal LN (popLN), which is located distally to the injury site, in the popliteal fossa underneath the knee (Fig. 1 a). Nerve transection induced a high amount of nodal expansion, with cellularity peaking 2 wk after axotomy, while leaving the contralateral side completely unaffected (Fig. 1, b and c; and Fig. S1 a). The surgery increased all leukocyte subsets assessed, and to a lesser extent the stromal cell compartment (Fig. S1 b), but most strongly affected B lymphocyte numbers (Fig. 1 c). To investigate which nerve branch was responsible, we unilaterally cut the sciatic, the femoral, or both nerves and assessed popLN cellularity 2 wk after surgery. Interestingly, denervation of the sciatic nerve by itself completely reproduced the phenotype caused by cutting both nerves, whereas femoral nerve transection alone had little effect on popLN cellularity (Fig. 1 d). We could replicate this effect with a nonsurgical method by specifically ablating sciatic nerve function chemically via local application of phenol, a treatment that provides a temporal nerve block (Sung et al., 2001), confirming that loss of neural function produced the phenotype and not the general surgery per se (Fig. S1 c). Resection of the sciatic nerve prevents viral spread from the popLN to the central nervous system (Iannacone et al., 2010), indicating that the popLN is directly innervated by the sciatic nerve. To examine whether nodal expansion was due to loss of direct innervation, sciatic axotomy was performed at the ankle level, distal to and thus bypassing the node (Fig. 1 e). This surgery produced little LN expansion compared with the control side (Fig. 1 f), demonstrating that loss of direct innervation to the popLN was critical for the phenotype.

In line with these data, nodal expansion was not simply an inflammatory response to a peripheral nerve injury—a process known as Wallerian degeneration, which also occurs after femoral nerve axotomy (Rotshenker, 2011)—since transplanting the sectioned sciatic nerve segment from the denervated to the nerve-intact side did not cause popLN expansion in the latter (Fig. 1, g and h). While we did observe some inflammation in the muscle surrounding the popLN, levels were similar in both femoral nerve denervation and sciatic nerve denervation conditions (Fig. S1, d and e), indicating that this could not account for the difference in LN size. Although in both single nerve denervation scenarios, general mobility of the animal remained largely normal, we investigated whether denervation had potential effects on the regional circulation. However, superficial blood flow in the leg was not altered by surgery (Fig. S1 f). In addition, accumulation of lymph in the draining popLN was unchanged between the nerve-lesioned and intact sides (Fig. S1 g), indicating the phenotype was not caused by gross alterations in regional blood flow or lymph percolation. These observations were further strengthened by data showing that in these experiments, the inguinal LN, which is not innervated by the sciatic nerve, yet drains lymph from the efferent lymphatics of the popLN, did not exhibit hypercellularity (Fig. S1 h). Together, these data demonstrate that loss of direct, sciatic innervation to the popLN triggers nodal expansion.

### LN expansion requires input from blood and afferent lymph

**Figure 2.**
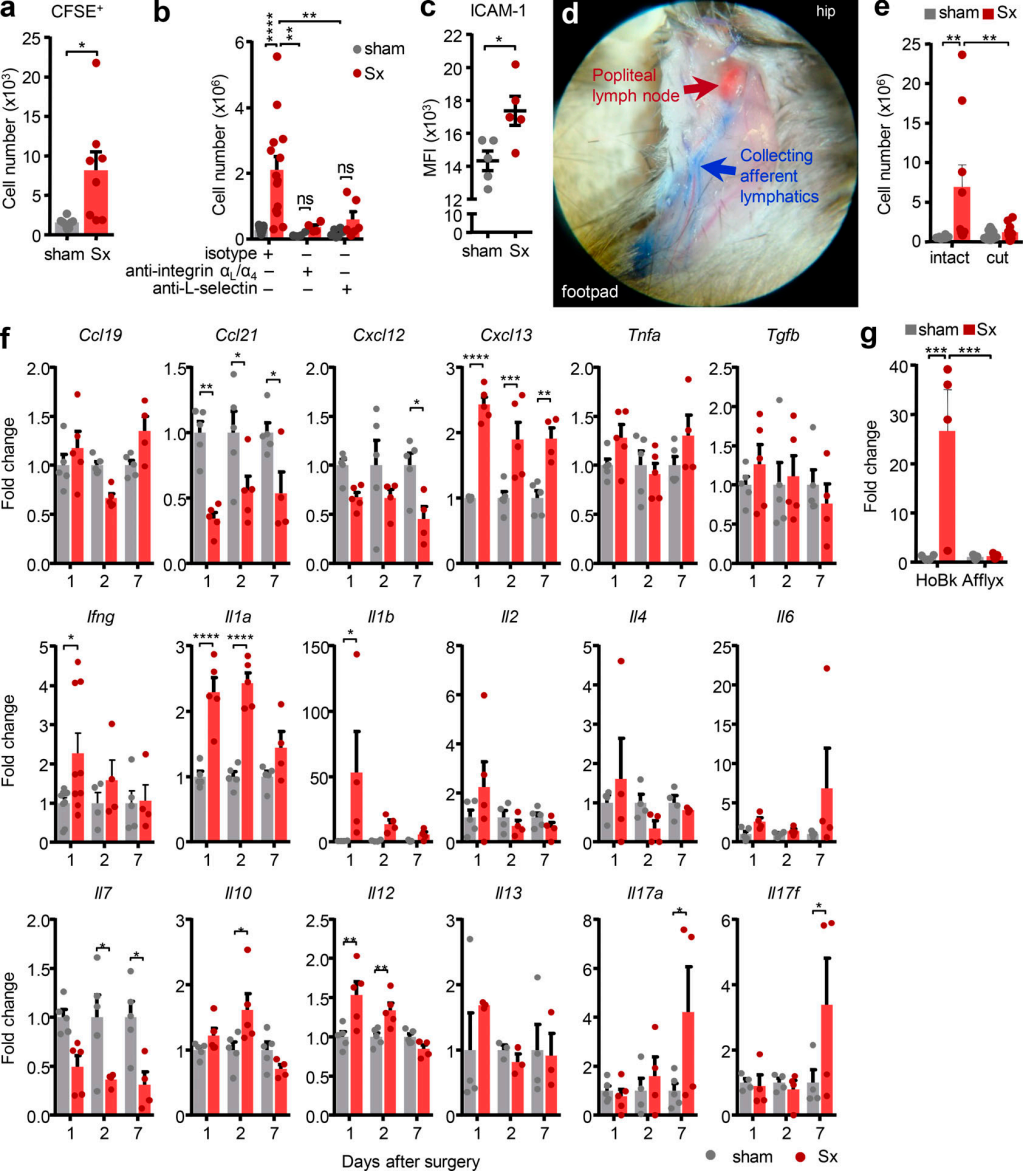
**Sciatic denervation creates an inflammatory milieu in the popLN. (a)** Quantification of CFSE^+^ transferred cells in popLNs a week after unilateral sciatic denervation; *n* = 8 mice from two independent experiments, unpaired Student’s *t* test. **(b)** Cellularity of the popLN after treatment with homing blocking antibodies a week after unilateral sciatic denervation; *n* = 4–15 mice from three independent experiments, two-way ANOVA, Šídák’s post-test. **(c)** Expression of ICAM-1 on HEVs in sham-operated or denervated popLNs 2 wk after unilateral sciatic denervation; *n* = 5 mice; data are representative of two independent experiments; unpaired Student’s *t* test. **(d)** Example of surgery to disconnect afferent lymphatic input to the popLN. **(e)** Cellularity of the popLN with or without afferent lymphatic disconnection a week after unilateral sciatic denervation; *n* = 8–13 mice from three independent experiments; two-way ANOVA; Šídák’s post-test. **(f)** Time course of the cytokine expression profile by qPCR of whole popLNs after unilateral sciatic denervation; *n* = 5–9 mice; data are representative of two independent experiments; one-way ANOVA; Tukey’s post-test. **(g)** Quantification of *Il1b* mRNA in the popLN 1 d after unilateral sciatic denervation with L-selectin homing blockade (HoBk) or afferent lymphatic disconnection (Afflyx); *n* = 4 or 5 mice from two independent experiments; two-way ANOVA; Šídák’s post-test. All mice in this figure were 8–12-wk-old WT males purchased from Charles River or Janvier Labs. All data are presented as mean ± SEM; *, P < 0.05; **, P < 0.01; ***, P < 0.001; ****, P < 0.0001. MFI, mean fluorescence intensity; Sx, sciatic denervation.

**Figure S2.**
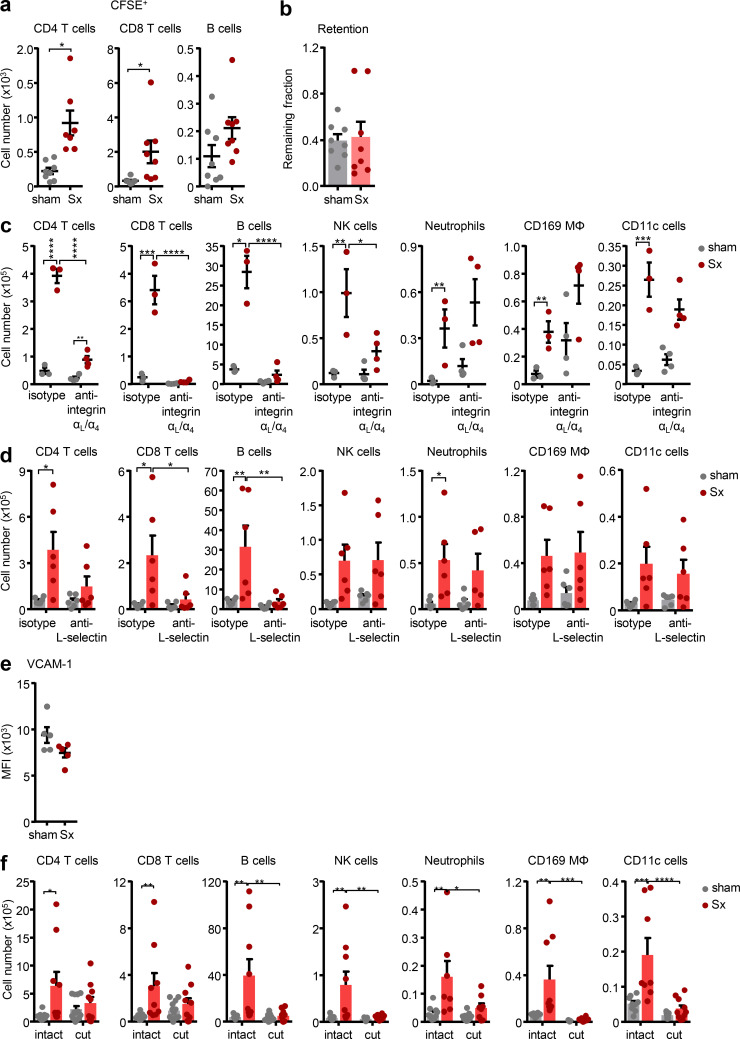
**LN expansion requires input from blood and afferent lymph. (a)** CFSE^+^ transferred T and B cells in popLN 1 wk after unilateral sciatic denervation; *n* = 8 mice from two independent experiments; unpaired Student’s *t* test. **(b)** Remaining fraction of adoptively transferred, labeled cells in intact or denervated popLNs after 18 h of homing blockade; *n* = 8 mice from two independent experiments. **(c, d, and f)** Counts of indicated subsets in popLNs 1 wk after unilateral sciatic denervation with or without (c) anti-α_L_/α_4_-integrin treatment (*n* = 3 or 4 mice; data are representative of two independent experiments), (d) anti–L-selectin treatment (*n* = 6 mice from two independent experiments), or (f) afferent lymphatic disconnection (*n* = 8–13 mice from three independent experiments); two-way ANOVA; Šídák’s post-test. **(e)** Expression of VCAM-1 on HEVs in sham-operated or denervated popLNs 2 wk after unilateral sciatic denervation; *n* = 5 mice; data are representative of two independent experiments; unpaired Student’s *t* test. All mice in this figure were 8–12-wk-old WT males purchased from Charles River or Janvier Labs. All data are presented as mean ± SEM; *, P < 0.05; **, P < 0.01; ***, P < 0.001; ****, P < 0.0001. Mϕ, macrophage; MFI, mean fluorescence intensity; Sx, sciatic denervation.

Since the dominant effect was observed in an increase of leukocyte populations, we first focused on whether nerve resection enhanced leukocyte trafficking to the popLN from blood. I.v. adoptive transfer of leukocytes harvested from untreated control mice to unilaterally nerve-operated animals revealed a strong increase in the ability of the denervated popLN to recruit leukocytes, predominantly T lymphocytes, compared with the sham-operated control side (Fig. 2 a and Fig. S2 a), while retention of cells was not affected (Fig. S2 b). This enhanced recruitment was critical for the overall phenotype, since blocking cellular input to the LN with antibodies directed against α_L_- and α_4_-integrins or L-selectin, molecules required for LN homing (von Andrian and Mempel, 2003), abolished LN expansion (Fig. 2 b; and Fig. S2, c and d). This indicated both ICAM-1 and VCAM-1, the counter-receptors for α_L_- and α_4_-integrins, to be implicated in the process, and we observed an up-regulation of ICAM-1 (but not VCAM-1) on high endothelial venules (HEVs; Fig. 2 c and Fig. S2 e). Blocking leukocyte recruitment greatly reduced overall LN cellularity after denervation, mainly by decreasing lymphocyte populations, including B cells and CD4 and CD8 T cells, demonstrating these cells to be the main contributors to LN expansion (Fig. S2, c and d). Conversely, the treatment had no effect on the increase in neutrophils, macrophages, and dendritic cells (DCs). Neutrophils are almost entirely prevented from entering the LN from blood after anti-integrin or –L-selectin treatment (Bogoslowski et al., 2020; He et al., 2018), whereas macrophages and conventional DCs generally do not enter the LN via this route (Girard et al., 2012; Jackson, 2019). This indicated that lymphocytes reached the LN via immigration from blood after sciatic nerve transection. In contrast, other leukocytes subsets likely increased within the LN due to local proliferation or input via afferent lymph (Girard et al., 2012).

Hence, we assessed the influence of afferent lymphatics on LN cellularity after nerve surgery. We microsurgically disconnected the popLN from afferent lymph by transecting all afferent lymphatic vessels draining to the node (Hendriks, 1978). This surgery completely abrogated the accumulation of dye in the popLN after intraplantar injection, confirming the successful operation (Fig. 2 d). Of importance, transection of afferent lymphatics to the popLN in both sides, to control for potential effects of this surgery (Hendriks et al., 1980; Hendriks et al., 1987; Hoshi et al., 1985), followed by unilateral nerve transection, entirely ablated the increase in LN cellularity in the denervated side only (Fig. 2 e and Fig. S2 f). This indicated that input from afferent lymphatics was important for the response. Together, this demonstrated that loss of innervation to the popLN induces lymphocyte recruitment from blood, which, together with input from afferent lymph, is a prerequisite for LN expansion in this setting.

### Sciatic nerve axotomy causes a pro-inflammatory milieu in the LN

These data indicated the existence of a pro-inflammatory milieu within the denervated LN. Indeed, quantitative PCR (qPCR) analyses of denervated and innervated contralateral control popLNs at different time points after surgery demonstrated a significant increase in several cytokines in the denervated side, including *Ifng*, *Il1a*, *Il1b*, *Il10*, *Il12*, *Il17a*, and *Il17f*, and the chemokine *Cxcl13* (Fig. 2 f). To unravel the etiology of this cytokine profile, we blocked either leukocyte homing from blood or any input to the LN via afferent lymph and assessed levels of *Il1b* as proxy for inflammation, due to its strong induction after nerve surgery. Interestingly, blocking homing from blood had no effect on the increase in *Il1b* expression, whereas induction was completely abrogated when afferent lymphatics were disconnected (Fig. 2 g). This demonstrated that the increase in *Il1b* was not dependent on cellular infiltration to the LN from blood but was a response to input reaching the LN via afferent lymph, indicating the latter to be located upstream in the chain of events.

### Loss of neural tone causes an autoimmune response to peripheral nerve antigens

**Figure S3.**
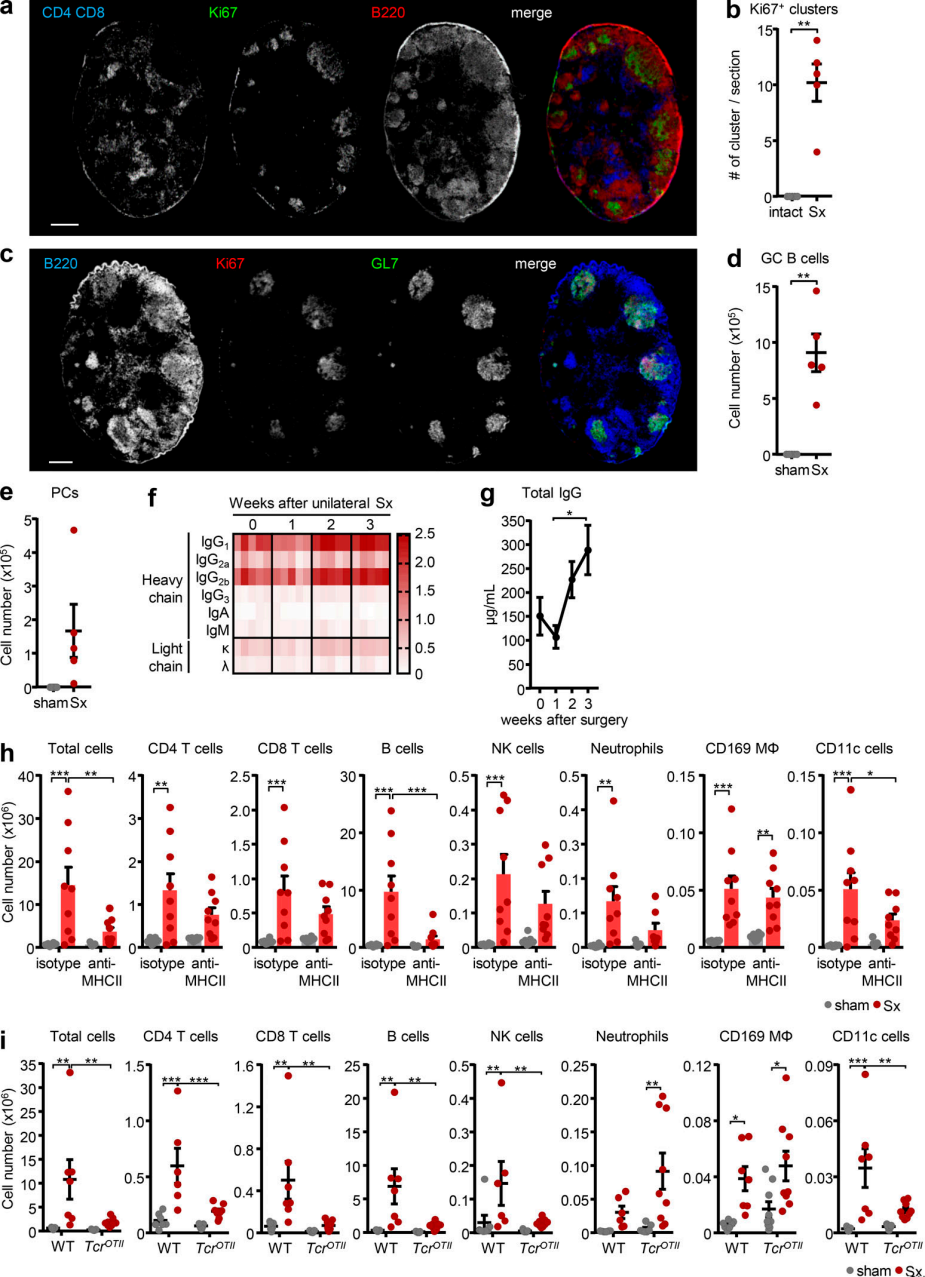
**Sciatic denervation drives a B cell response and IgG production. (a)** Identification of proliferating cells using T cell (CD4 plus CD8), B cell (B220), and proliferation (Ki67) markers in denervated popLNs, 2 wk after unilateral sciatic denervation. Scale bar, 300 µm. Images are representative of two independent experiments. **(b)** Quantification of proliferating clusters in denervated popLNs, 2 wk after unilateral sciatic denervation; *n* = 5 mice; data are representative of two independent experiments; unpaired Student’s *t* test. **(c)** Characterization of proliferating cell clusters using B cell (B220), proliferation (Ki67), and GC (GL7) markers in denervated popLNs, 2 wk after unilateral sciatic denervation. Scale bar, 300 µm. Images are representative of two independent experiments. **(d and e)** Quantification of GC B cells (d) and plasma cells (PCs; e) in popLNs, 3 wk after unilateral sciatic denervation; *n* = 5 mice; data are representative of two independent experiments; unpaired Student’s *t* test. **(f)** Isotyping of mouse sera plotted as increased light absorption units and **(g)** quantification of total serum IgG levels at indicated time points after unilateral sciatic denervation; *n* = 5 mice; data are representative of two independent experiments; one-way ANOVA; Tukey’s post-test. **(h)** Cellularity of popLNs and counts of indicated subsets a week after unilateral sciatic denervation with or without anti-MHCII antibody treatment; *n* = 8 or 9 mice from three independent experiments; two-way ANOVA; Šídák’s post-test. **(i)** Cellularity of popLNs and counts of indicated subsets in WT or *Tcr^OTII^* mice 1 wk after unilateral sciatic denervation; *n* = 7–9 mice from three independent experiments; two-way ANOVA; Šídák’s post-test. *Tcr^OTII^* mice were 9–20 wk old of both sexes and bred in the animal facility. Age- and sex-matched WT control mice were purchased from Janvier Labs and housed in the same facility with *Tcr^OTII^* mice for a week before experimentation. All other mice in this figure were 8–12-wk-old WT males purchased from Charles River or Janvier Labs. All data are presented as mean ± SEM; *, P < 0.05; **, P < 0.01; ***, P < 0.001. Mig, migratory DC; Res, resident DC; Sx, sciatic denervation.

While the initial increase in LN cellularity was due to leukocyte infiltration (Fig. 2 a), 2 wk after the surgery, we observed a strong increase in proliferation, as assessed by Ki67 staining, which was restricted to B cell follicles (Fig. S3, a and b). Proliferative B cells were positive for the activation marker GL7, classifying them as germinal center (GC) B cells (Fig. S3, c and d). Since the generation of GCs requires engagement between CD4 T cells and B cells (Jones and Janeway, 1981; Okada et al., 2005), this demonstrated the reaction to be a T cell–dependent B cell response, leading to terminal differentiation of B cells into plasma cells. Indeed, we observed expansion of GC B cells and plasma cells in the popLN, which we failed to identify under steady-state conditions (Fig. S3, d and e), and enhanced IgG1 and IgG2b but not IgM antibody levels in serum (Fig. S3, f and g). This furthermore denoted the requirement of antigen and TCR–MHCII engagement (Germain, 1994). Indeed, blockade with an antibody directed against the MHCII complex to curb antigen presentation ablated the LN response, an effect most prominently observed for B cell numbers (Fig. S3 h). Furthermore, denervation surgeries in *Tcr^OTII^* mice, which exhibit a greatly restricted CD4 TCR repertoire, predominantly directed against ovalbumin (Barnden et al., 1998), did not cause comparable levels of nodal expansion (Fig. S3 i). This indicated that sufficient numbers of cognate CD4 T cells and their interactions with the MHCII complex were required for mounting a response after nerve injury. Together, these data demonstrated the importance of antigen and CD4 T cells in the LN response, which at later stages was dominated by B cell proliferation, GC formation, and plasma cell differentiation.

**Figure S4.**
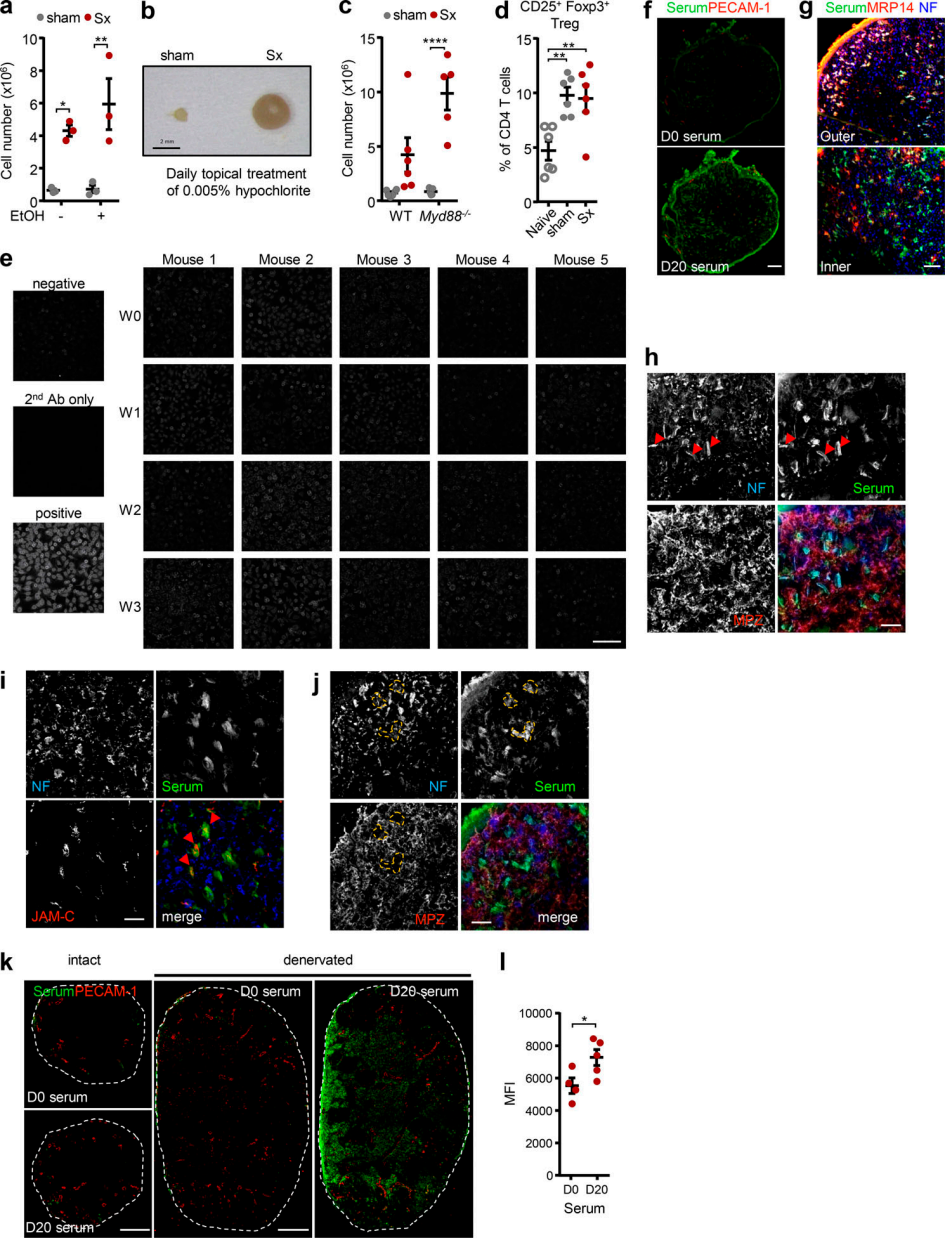
**Sciatic denervation induces an autoimmune response to peripheral nerve antigens. (a)** Cellularity of popLNs 1 wk after unilateral sciatic denervation with or without broader ethanol disinfection; *n* = 3 mice; data are representative of two independent experiments; two-way ANOVA; Šídák’s post-test. **(b)** An image of popLNs 1 wk after unilateral sciatic denervation with daily topical hypochlorite treatment. **(c)** Cellularity of popLNs in WT or Myd88-KO mice a week after unilateral sciatic denervation. *n* = 5 or 6 mice; data are representative of two independent experiments; two-way ANOVA; Šídák’s post-test. **(d)** Quantification of CD25^+^ Foxp3^+^ regulatory T cells in the naive, sham-operated, or denervated popLNs 7 d after surgery; *n* = 6 from two independent experiments; one-way ANOVA; Tukey’s post-test. **(e)** Human epithelial cell (HEp2) slides incubated with sera generated from different time points after unilateral sciatic denervation. Scale bar, 200 µm. **(f)** Immunoreactivity of serum (green) harvested from mice 0 or 20 d after unilateral sciatic denervation to the intact sciatic nerve. Sections were costained with antibodies directed against CD31 (red). Scale bar, 100 μm. Images are representative of two independent experiments. **(g)** Immunoreactivity of serum (green) harvested from mice 20 d after unilateral sciatic denervation to the intact sciatic nerve. Sections were costained with antibodies directed against MRP14 (red) and neurofilament (NF; blue). Scale bar, 30 μm. Images are representative of two independent experiments. **(h and j)** Staining of serum harvested from mice 20 d after unilateral sciatic denervation on the sciatic nerve together with antibodies directed against NF and MPZ. Scale bars, 20 µm. **(i and k)** Immunoreactivity of serum harvested from mice after unilateral sciatic denervation to the sciatic nerve (i) and popLNs (k). Sections were costained with antibodies directed against CD31 (vasculature), MRP-14 (Schwann cell), NF (nerve), or JAM-C (noncompact myelin). Scale bars, 20 µm (i); and 200 (intact) and 300 (denervated; j) µm. Images are representative of two independent experiments. **(l)** Quantification of immunoreactivity of sera harvested from mice and normalized to total IgG levels after unilateral sciatic denervation to intact and denervated popLN from before (D0) or 3 wk (D20) after unilateral sciatic denervation on popLNs; *n* = 4 or 5 mice; data are representative of two independent experiments; two-way ANOVA; Šídák’s post-test. *Myd88^−/−^* mice were 8–12 wk old and bred in the animal facility. Their age- and sex-matched controls and all other mice in this figure were 8–12-wk-old WT males purchased from Charles River or Janvier Labs. All data are presented as mean ± SEM; *, P < 0.05; **, P < 0.01; ****, P < 0.0001. Ab, antibody; MFI, mean fluorescence intensity; Sx, sciatic denervation; Treg, regulatory T cell; W, week.

To assess whether skin commensal microbes were involved in the response, we thoroughly disinfected the surgery site, the legs, and the flank of the mouse in a daily manner. However, this had no effect on the immune response (Fig. S4, a and b). Furthermore, the lack of an effect in mice deficient in *Myd88*, a critical signaling component in the detection of microbial factors (Fig. S4 c), indicated that the response was caused by a sterile injury and not by a potential environmental barrier breakdown and associated influx of microbial antigens. Furthermore, the response did not appear to be due to loss of peripheral tolerance to antigen, since percentages of regulatory T cells did not change in the denervated popLN (Fig. S4 d).

However, denervation-induced antibodies did not target common nuclear auto-antigens as expressed by HEp2 slides that are often used in the detection of various autoimmune diseases (Fig. S4 e; Damoiseaux et al., 2019). In contrast, staining of sciatic nerve sections with serum obtained from mice without or 20 d after sciatic nerve surgery showed a strong immunoreactivity (Fig. S4 f). Specifically, we detected staining of Schwann cells (Fig. S4 g) and, to a lesser extent, nerves (Fig. S4 h). In Schwann cells, staining was predominantly observed in regions of noncompact (Fig. S4 i) but not compact myelin (Fig. S4 j), as assessed with the respective markers JAM-C and myelin protein zero (MPZ; Scheiermann et al., 2007). Furthermore, the antiserum showed strong immunoreactivity in denervated but not contra-lateral intact popLNs, indicating that self-antigens were released locally and present within the draining LN (Fig. S4, k and l). Together, these observations demonstrated that sciatic nerve injury caused an immune response directed against peripheral nerve autoantigens.

**Figure 3.**
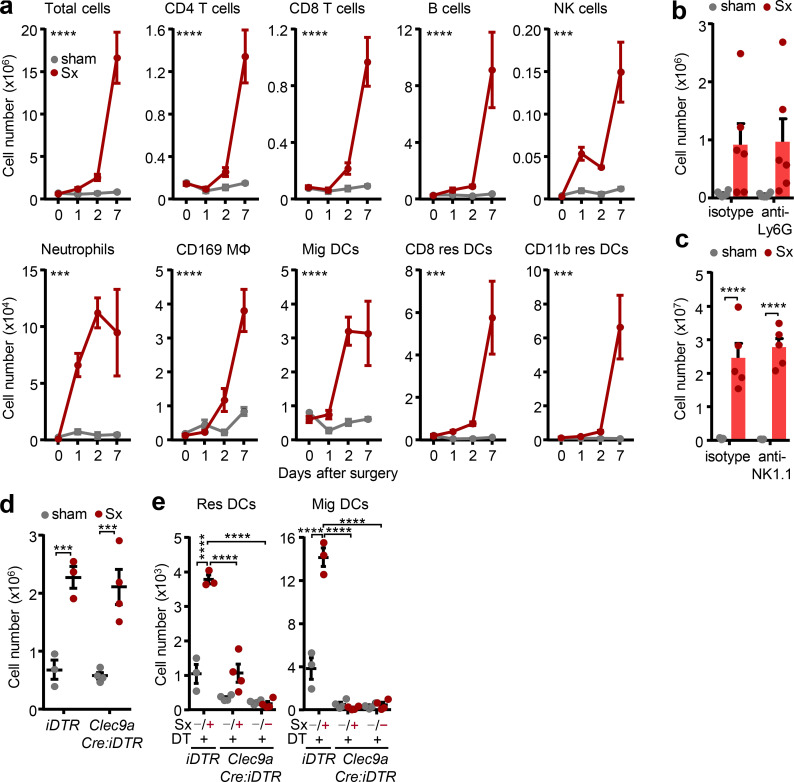
**Involvement of different leukocyte subsets. (a)** Time course (in days) of leukocyte subsets in sham-operated and denervated popLNs after unilateral surgical denervation; *n* = 3–5 mice from two independent experiments; two-way ANOVA; Šídák’s post-test. Asterisks indicate significance between sham-operated and denervated popLNs. **(b and c)** Cellularity of popLNs 1 wk after unilateral sciatic denervation in combination with antibodies to deplete neutrophils (anti-Ly6G; *n* = 6 mice from two independent experiments; b), or NK cells (anti-NK1.1; *n* = 5 mice from two independent experiments; c); two-way ANOVA; Šídák’s post-test. **(d)** Cellularity of popLNs in diphtheria toxin–treated *Clec9aCre:Rosa-iDTR* mice or control *iDTR* mice 2 d after unilateral sciatic denervation; *n* = 3 or 4 mice; data are representative of two independent experiments; two-way ANOVA; Šídák’s post-test. **(e)** Counts of resident and migratory DCs in popLNs in diphtheria toxin–treated *Clec9aCre:Rosa-iDTR* or control mice 2 d after unilateral sciatic denervation; *n* = 3 or 4 mice; data are representative of two independent experiments; two-way ANOVA; Šídák’s post-test. *Clec9aCre:Rosa-iDTR* and *iDTR* mice were 8–12 wk old and bred in our animal facility. The other mice in this figure were 8–12-wk-old WT males purchased from Charles River or Janvier Labs. All data are presented as mean ± SEM; ***, P < 0.001; ****, P < 0.0001. DT, diphtheria toxin; Mig, migratory DC; Res, resident DC; Sx, sciatic denervation.

**Figure S5.**
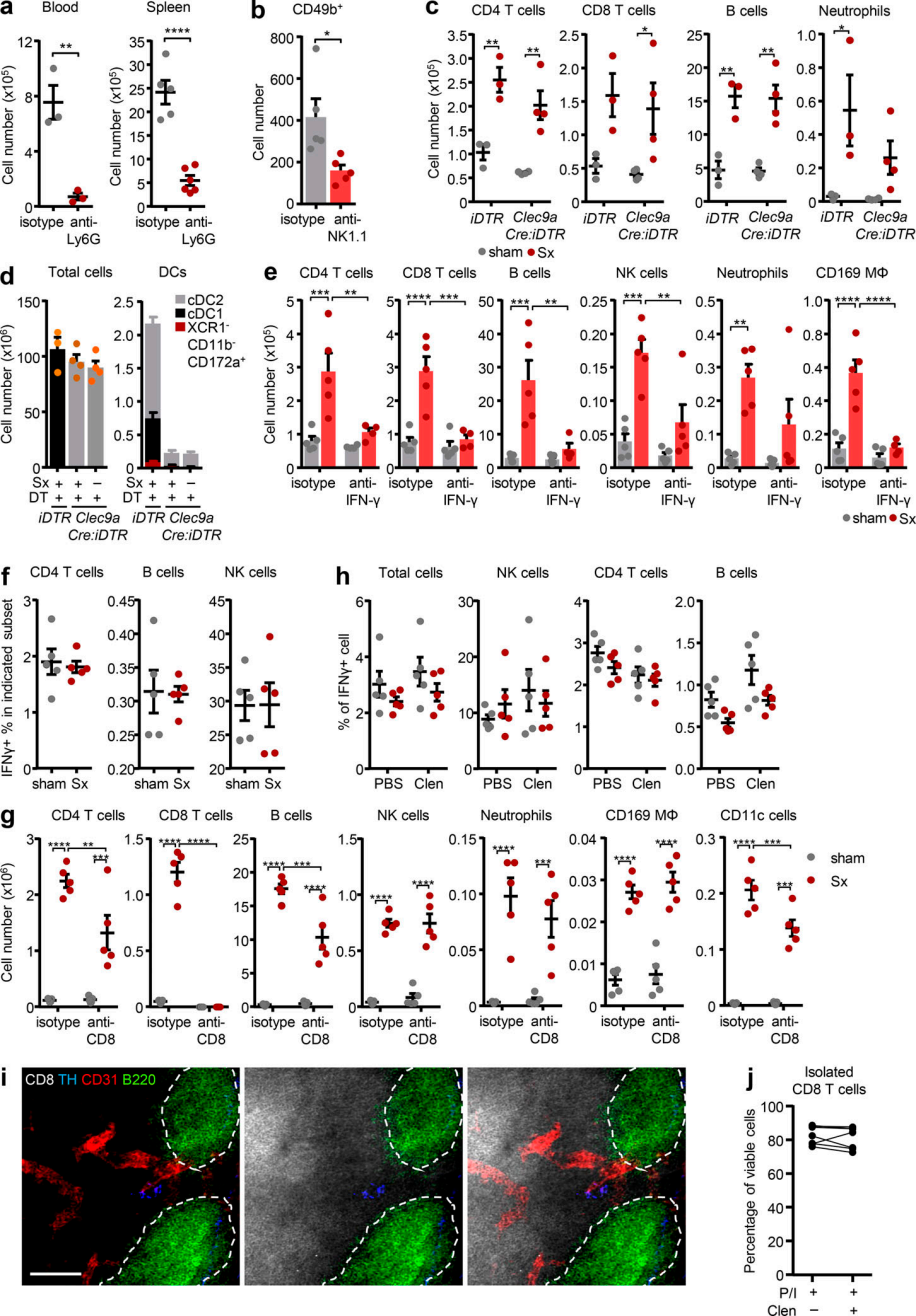
**IFN-γ expression and CD8 T cells drive LN expansion. (a and b)** Efficacy of anti-Ly6G antibody-mediated neutrophil depletion (*n* = 3–6 mice from two independent experiments; a) or anti-NK1.1 antibody-mediated NK cell depletion (*n* = 5 mice from two independent experiments; b); unpaired Student’s *t* test. **(c)** Counts of lymphocytes and neutrophils in popLNs in diphtheria toxin–treated *Clec9aCre:Rosa-iDTR* or *iDTR* mice 2 d after unilateral sciatic denervation; *n* = 3–4 mice; data are representative of two independent experiments; two-way ANOVA; Šídák’s post-test. **(d)** Efficacy of diphtheria toxin–mediated *Clec9aCre:Rosa-iDTR*–specific cell depletion. Counts of total cells and DC subsets in spleen in diphtheria toxin–treated *Clec9aCre:Rosa-iDTR* or control mice 2 d after unilateral sciatic denervation; *n* = 3–4 mice; data are representative of two independent experiments; one-way ANOVA; Tukey’s post-test. **(e)** Counts of indicated subsets in popLNs 1 wk after unilateral sciatic denervation with or without IFN-γ blockade; *n* = 5 mice; data are representative of two independent experiments; two-way ANOVA; Šídák’s post-test. **(f)** Percentage of IFN-γ^+^ cells in indicated subsets; *n* = 5 mice; data are representative of two independent experiments; unpaired Student’s *t* test. **(g)** Counts of indicated subsets in popLNs 1 wk after unilateral sciatic denervation with or without CD8 depletion; *n* = 5 mice; data are representative of two independent experiments; two-way ANOVA; Šídák’s post-test. **(h)** Percentage of IFN-γ^+^ cells in indicated subsets from popLNs 1 wk after unilateral sciatic denervation with PBS or clenbuterol treatment; *n* = 5 mice; data are representative of two independent experiments; two-way ANOVA; Šídák’s post-test. **(i)** Presence of sympathetic TH^+^ nerves close to CD8 T cells in the popLN. B cell follicles are highlighted with dotted lines. Scale bar, 50 µm. **(j)** Percentage of viable isolated CD8 T cells after in vitro restimulation with PMA/ionomycin (P/I) in the presence or absence of clenbuterol; *n* = 7 mice from two independent experiments. *Clec9aCre:Rosa-iDTR* and *iDTR* mice were 8–12 wk old and bred in the animal facility. The other mice in this figure were 8–12-wk-old WT males purchased from Charles River or Janvier Labs. All data are presented as mean ± SEM; *, P < 0.05; **, P < 0.01; ***, P < 0.001; ****, P < 0.0001. Clen, clenbuterol; DT, diphtheria toxin; Mϕ, macrophage; Sx, sciatic denervation.

We next focused on the leukocyte subset(s) governing the reaction and screened the early time points after nerve surgery for the appearance of specific leukocyte subsets in the LN. CD4 and CD8 T lymphocytes as well as B cells markedly increased on day 7 (D7), along with macrophages and resident DCs (Fig. 3 a). In contrast, neutrophils, NK cells, and migratory DCs showed increases as early as 1–2 d after surgery (Fig. 3 a), implying a potential initiator role for these cell types. Additionally, these latter cell types were not recruited from blood, a step that was likely occurring at a later time point, as pharmacological homing blockade had no effects on their numbers in the LN (Fig. S2, c and d). However, neither antibody-mediated depletion of neutrophils nor a reduction in NK cell numbers exhibited an effect on LN expansion, designating them to be bystander cells in this scenario (Fig. 3, b and c; and Fig. S5, a and b). We next investigated the role of DCs as antigen presenting cells draining peripheral sites. However, depleting DCs genetically using *Clec9aCre:Rosa-iDTR* mice (Schraml et al., 2013) had no effect on LN cellularity (Fig. 3 d and Fig. S5 c), although numbers of migratory DCs were abolished and resident DCs were strongly reduced (Fig. 3 e and Fig. S5 d). This indicated other cell types to be involved in the response.

### IFN-γ expression and CD8 T cells drive LN expansion

**Figure 4.**
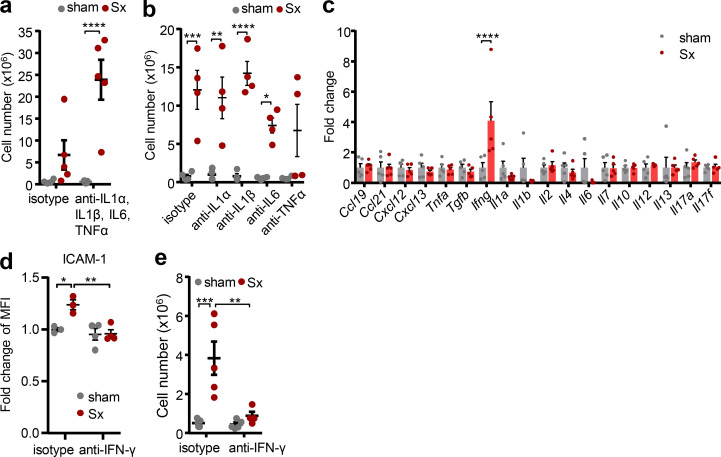
**IFN-γ is required for LN expansion. (a and b)** Cellularity of popLNs 1 wk after unilateral sciatic denervation with antibodies directed against IL-1α, IL-1β, IL-6, and/or TNF-α in combination (*n* = 5 mice; a) or individually (*n* = 4 mice; b); data are representative of two independent experiments; two-way ANOVA; Šídák’s post-test. **(c)** Cytokine expression profile of isolated (homing block plus disconnected afferent lymphatics on both legs) popLNs 2 d after unilateral sciatic denervation; *n* = 5 mice; data are representative of two independent experiments; two-way ANOVA; Šídák’s post-test. **(d)** Expression of ICAM-1 on HEVs in sham-operated or denervated popLNs 1 wk after unilateral sciatic denervation with or without IFN-γ blockade; *n* = 3 or 4 mice; data are representative of two independent experiments; two-way ANOVA; Šídák’s post-test. **(e)** Cellularity of the popLN 1 wk after unilateral sciatic denervation with or without IFN-γ blockade; *n* = 5 mice; data are representative of two independent experiments; two-way ANOVA; Šídák’s post-test. All mice in this figure were 8–12-wk-old WT males purchased from Charles River or Janvier Labs. All data are presented as mean ± SEM; *, P < 0.05; **, P < 0.01; ***, P < 0.001; ****, P < 0.0001. MFI, mean fluorescence intensity; Sx, sciatic denervation.

**Figure 5.**
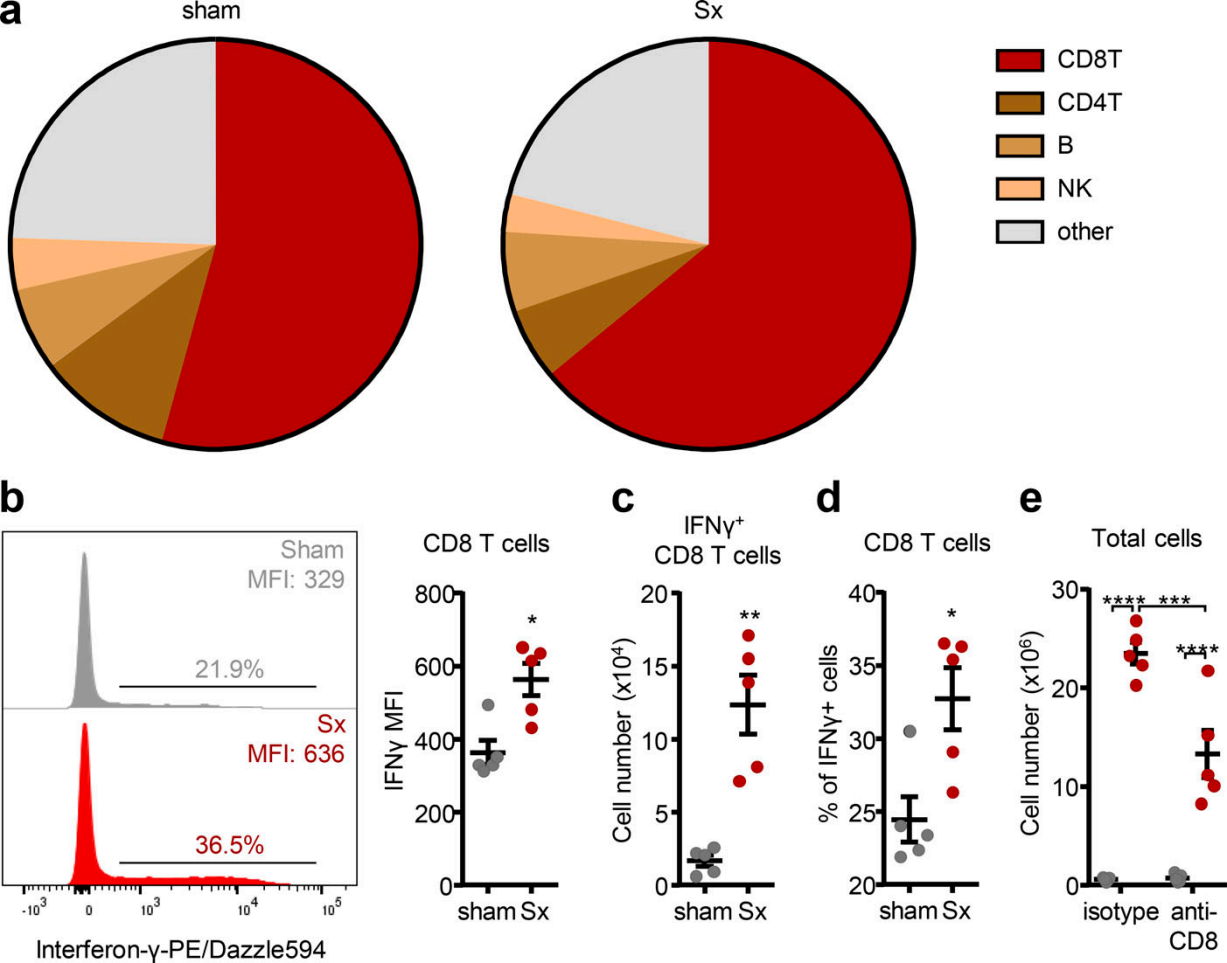
**CD8 T cells are the major producers of IFN-γ*.* (a)** Subset composition of IFN-γ–expressing cells in the intact or denervated popLN. Data are presented as the mean of five mice; data are representative of two independent experiments. **(b)** Representative histogram and quantified geometric mean fluorescence intensity of IFN-γ in CD8 T cells; *n* = 5 mice; data are representative of two independent experiments; unpaired Student’s *t* test. **(c and d)** Cell number (c) and percentage (d) of IFN-γ–expressing cells in CD8 T cells; *n* = 5 mice; data are representative of two independent experiments; unpaired Student’s *t* test. **(e)** Cellularity of the popLN 1 wk after unilateral sciatic denervation with or without CD8 depletion; *n* = 5 mice; data are representative of two independent experiments; two-way ANOVA; Šídák’s post-test. All mice in this figure were 8–12-wk-old WT males purchased from Charles River or Janvier Labs. All data are presented as mean ± SEM; *, P < 0.05; **, P < 0.01; ***, P < 0.001; ****, P < 0.0001. MFI, mean fluorescence intensity; Sx, sciatic denervation.

To identify the relevant cell type, we investigated the effect of loss of direct innervation on LN function in mechanistic detail. Surprisingly, given the strong increase of *Il1b* (Fig. 2 f), blocking the pro-inflammatory cytokines IL-1α, IL-1β, and IL-6 as well as TNF-α, individually or in combination, had no effect on LN cellularity (Fig. 4, a and b). In line with this, the pro-inflammatory profile was not a response to a potential danger signal, as surgeries performed in *Myd88*-deficient animals, a key downstream signaling molecule for the IL-1 receptor pathway as well as for damage-associated molecular patterns (O’Neill and Bowie, 2007), did not alleviate LN expansion (Fig. S4 c). These data demonstrated that the highly pro-inflammatory milieu in denervated LNs was associated with but did not directly cause the phenotype. We thus investigated the effect of loss of direct neural tone on the LN itself, which might be masked by inflammatory signals reaching the LN from the blood or its drainage area. We pharmacologically and surgically isolated the popLN from any potential input reaching it via blood or afferent lymph by blocking leukocyte homing in combination with a disconnection of afferent lymphatics at the same time, and transected the sciatic nerve unilaterally. Unexpectedly, in this scenario, expression of only one cytokine, *Ifng*, was strongly induced in the nerve-resected side compared with the sham control side (Fig. 4 c). This increase in *Ifng* was of functional relevance, since blocking IFN-γ with antibodies ablated the increase in ICAM-1 expression on HEVs (Fig. 4 d) and completely inhibited LN expansion after nerve surgery (Fig. 4 e and Fig. S5 e). To examine which cell was producing the cytokine at the protein level, we performed flow cytometry analysis for intracellular IFN-γ of popLNs after sciatic nerve injury. CD8 T cells were the dominant IFN-γ–expressing cell type in the LN (Fig. 5, a–d; and Fig. S5 f). Furthermore, this cell type was critical to the response since depletion of CD8 T cells dampened LN expansion (Fig. 5 e and Fig. S5 g). These data demonstrated that loss of direct innervation to the LN had an impact on LN reactivity itself by increasing IFN-γ production in CD8 T cells, which was functionally involved in the ensuing expansion.

### Sympathetic but not sensory neural tone curbs LN responses

**Figure 6.**
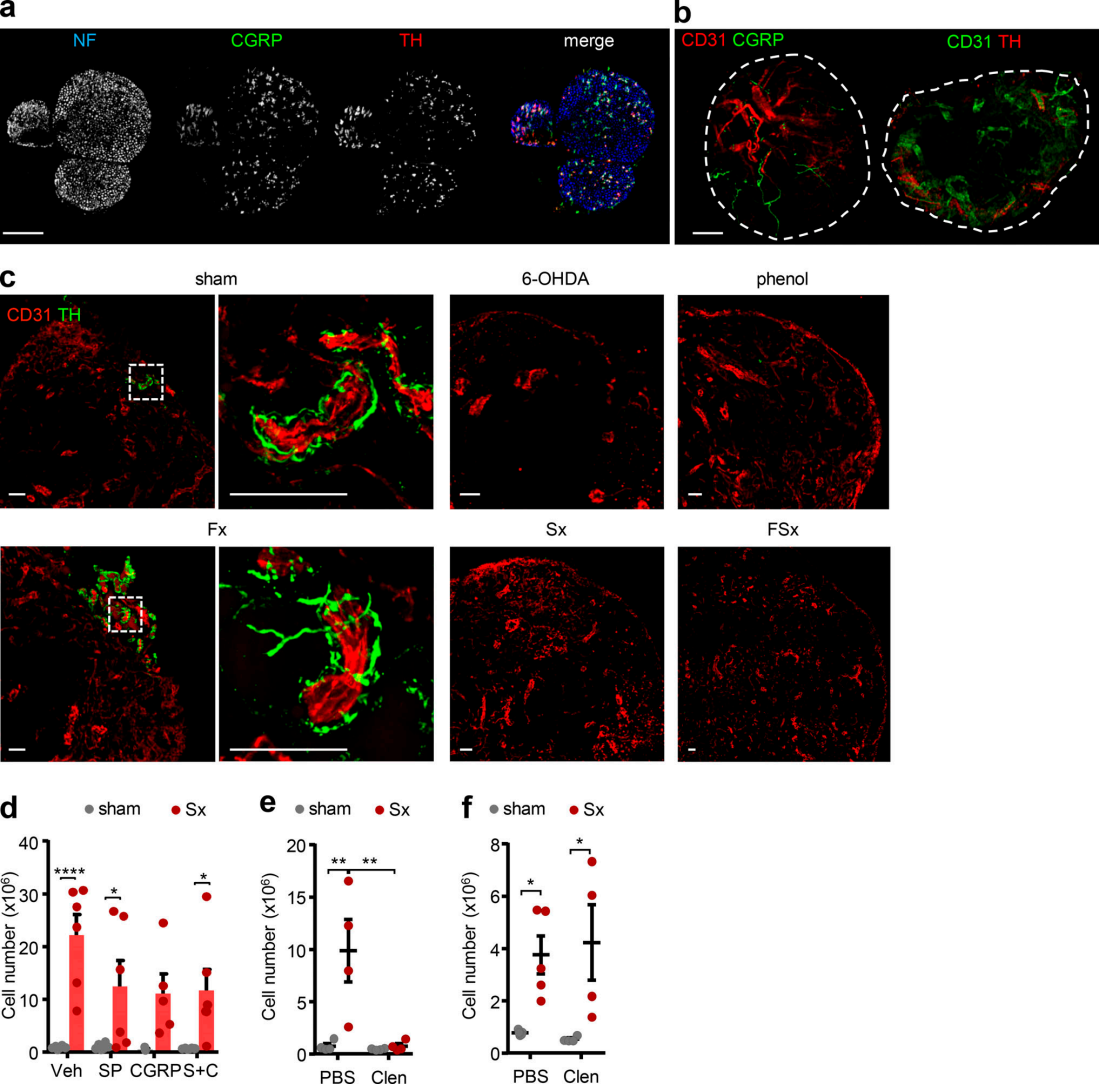
**Sympathetic nerves curb LN responses. (a)** Identification of sympathetic (red, TH) and nociceptive sensory (green, CGRP) nerves together with neurofilament (blue, NF) in the sciatic nerve. Scale bar, 200 µm. **(b)** Innervation of nociceptive sensory (left) and sympathetic (right) nerves to the popLN (circled), costained with CD31. Scale bar, 200 µm. **(c)** Innervation of sympathetic nerves to the popLN in untreated sham conditions, after systemic 6-OHDA treatment, local phenol treatment, or femoral (Fx), sciatic (Sx) or femoral and sciatic dual denervation (FSx) surgeries. Scale bars, 50 µm. **(d)** Cellularity of popLNs 1 wk after unilateral sciatic denervation in combination with substance P (SP), CGRP, or combined treatment (S+C); *n* = 5 or 6 mice from two independent experiments; two-way ANOVA; Šídák’s post-test. **(e)** Cellularity of the popLN 1 wk after unilateral sciatic denervation in combination with PBS or clenbuterol treatment; *n* = 4 mice; data are representative of two independent experiments; two-way ANOVA; Šídák’s post-test. **(f)** Cellularity of the popLN in *Adrb2^−/−^* mice 1 wk after unilateral sciatic denervation in combination with PBS or clenbuterol treatment; *n* = 4 mice; data are representative of two independent experiments; two-way ANOVA; Šídák’s post-test. *Adrb2^−/−^* mice were 8–12-wk-old males and bred in our animal facility. All other mice in this figure were 8–12-wk-old WT males purchased from Charles River or Janvier Labs. All data are presented as mean ± SEM; *, P < 0.05; **, P < 0.01; ****, P < 0.0001. Clen, clenbuterol; Veh, vehicle.

While motor neurons make up only ∼5% of the sciatic nerve bundle, the nerve exhibits predominantly sensory (∼70%) as well as sympathetic (∼25%) fibers (Savastano et al., 2014; Schmalbruch, 1986), which we identified with their respective markers calcitonin gene-related peptide (CGRP) as well as tyrosine hydroxylase (TH; Fig. 6 a). Innervation of these neural subsets was found along the vasculature of the popLN, confirming that neural input can reach the node directly (Fig. 6 b). Treatment with 6-hydroxydopamine (6-OHDA), a sympathetic neurotoxin (Kostrzewa and Jacobowitz, 1974), or phenol as well as denervation of both the sciatic and femoral or just the sciatic nerve decreased nerve staining, while only cutting the femoral nerve had no effect on sympathetic innervation levels (Fig. 6 c). These data thus demonstrated the sciatic nerve to be responsible for LN innervation, providing an anatomical explanation with respect to the observed functional results (Fig. 1 d). To assess the influence of sympathetic and sensory neural tone in the scenario, we unilaterally denervated mice and treated animals with either the main sensory nerve peptides CGRP or substance P, alone or in combination, or the β2 adrenergic receptor (Adrb2) agonist clenbuterol. Interestingly, treatment with clenbuterol reduced cellularity in the denervated LN to sham levels, thus completely ablating the denervation phenotype, while the sensory nerve agonists exhibited partial effects, which, however, were not statistically significant (Fig. 6, d and e). The rescue effect of clenbuterol was specific for the Adrb2, since it did not occur in *Adrb2*-deficient mice (Fig. 6 f). These data thus implicated loss of sympathetic nervous tone and specifically the Adrb2 in LN inflammation.

**Figure 7.**
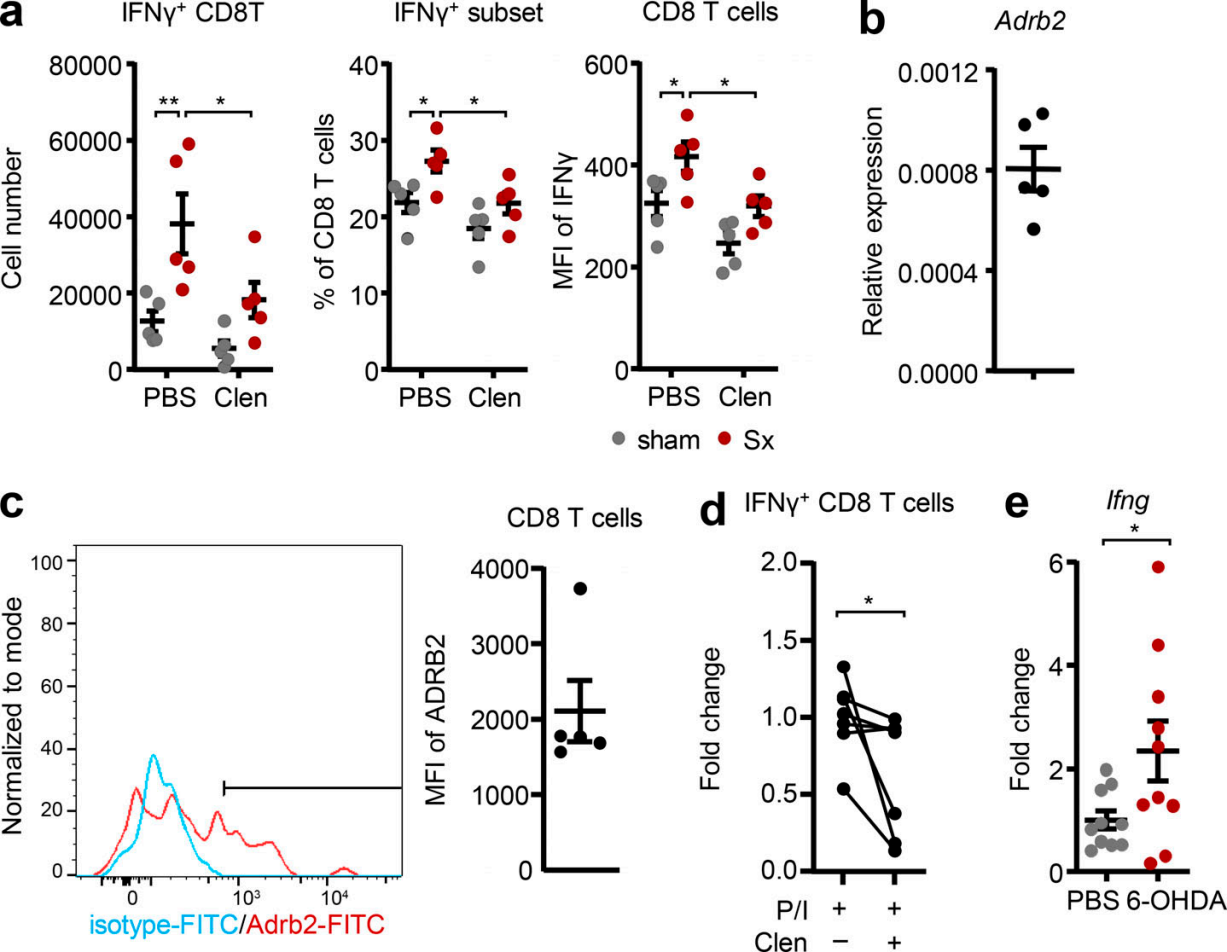
**β2 adrenergic signaling reduces IFN-γ expression in CD8 T cells. (a)** Cell number and percentage of IFN-γ^+^ CD8 T cells and geometric mean fluorescence intensity of IFN-γ staining in CD8 T cells; *n* = 4 mice; data are representative of two independent experiments; two-way ANOVA; Šídák’s post-test. **(b)** Expression of *Adrb2* in CD8 T cells in the popLN at steady-state by qPCR; *n* = 5 mice; data are representative of two independent experiments. **(c)** Expression of the Adrb2 on CD8 T cells in the popLN at steady-state by flow cytometry (blue: isotype; red: Adrb2 staining; the bar delineates specific staining); *n* = 5 mice; data are representative of two independent experiments. **(d)** Change in the percentage of IFN-γ^+^ cells in vitro after isolation of CD8 T cells and restimulation with PMA/ionomycin (P/I) in the presence or absence of clenbuterol; *n* = 7 mice from two independent experiments; Wilcoxon test. **(e)** Expression of *Ifng* in the popLN with or without chemical sympathectomy with 6-OHDA; *n* = 10 mice from two independent experiments; unpaired Student’s *t* test. All mice in this figure were 8–12-wk-old WT males purchased from Charles River or Janvier Labs. All data are presented as mean ± SEM; *, P < 0.05; **, P < 0.01. Clen, clenbuterol; MFI, mean fluorescence intensity; Sx, sciatic denervation.

Clenbuterol specifically reduced the numbers of IFN-γ^+^ CD8 T cells in the popLN, and reduced the percentage of IFN-γ–producing CD8 T cells as well as IFN-γ levels in these cells, while other cell types were not affected (Fig. 7 a and Fig. S5 h). Furthermore, we detected expression of the Adrb2 at both the mRNA and surface protein level in CD8 T cells, indicating that these cells can be direct targets of Adrb2-mediated sympathetic signals (Fig. 7, b and c). Indeed, sympathetic nerves were also seen in close proximity to CD8 T cells in the LN (Fig. S5 i), and clenbuterol treatment reduced IFN-γ levels of isolated CD8 T cells in vitro, without affecting their viability (Fig. 7 d and Fig. S5 j). Treatment with 6-OHDA increased levels of *Ifng* in popLN (Fig. 7 e), mimicking the response after sciatic denervation. Together, our data demonstrate that loss of local nerve-derived sympathetic, Adrb2-mediated signals increases IFN-γ production in popLN CD8 T cells after sciatic nerve injury and drives LN expansion.

## Discussion

We have shown here that loss of local innervation to the draining LN causes expansion in LN cellularity. Our data demonstrate that loss of sympathetic innervation to the popLN induces the expression of IFN-γ in CD8 T cells after sciatic nerve injury. IFN-γ production is of functional relevance for LN expansion, and this can be rescued via application of the Adrb2 agonist clenbuterol.

Although LNs in mice and humans have long been known to be innervated by the SNS (Felten et al., 1987; Felten et al., 1985; Felten et al., 1984; Sloan et al., 2007), the functional relevance has remained elusive. Our results show that loss of direct innervation to the LN causes a highly pro-inflammatory milieu that contributes to LN expansion after nerve injury. The data specifically implicate the sympathetic but not the sensory arm of the nervous system in this process.

Our data indicate that sympathectomy induces a functionally relevant increase of IFN-γ in popLNs, which could be mimicked by pharmacologically induced loss of sympathetic tone. This is reminiscent of a report in rats describing that acute surgical sympathetic denervation causes LNs to release increased amounts of IFN-γ (Castrillón et al., 2000). Systemic sympathectomy with 6-OHDA has been shown to enhance cell proliferation and leukocyte homing in the LN (Madden et al., 1994). Furthermore, 6-OHDA treatment in combination with influenza A virus infection was found to enhance CD8^+^ T cell proliferation and cytokine responses in the LN (Grebe et al., 2009). However, the mechanism for these responses had remained elusive. We show here that sympathectomy alters the LN microenvironmental milieu, which can prime lymphocytes for the encounter to antigen and subsequent proliferative responses. This observation suggests that tonic sympathetic signaling is an important factor in preventing exaggerated LN responses, asserting a predominantly anti-inflammatory role of the SNS in immunity. An important distinction has to be made that a major injury can result in an overreaction of the SNS at the systemic level (potentially also mediated by the adrenal gland) leading to reduced immunity and infections, while local loss of sympathetic innervation causes an increased immune response.

IFN-γ has been linked to autoimmunity, such as lupus development (Theofilopoulos et al., 2001). Genetic disruption of the IFN-γ receptor (IFN-γR) prevents the development of autoantibodies and kidney disease in lupus-prone mice (Haas et al., 1998; Schwarting et al., 1998). Furthermore, antibody treatment against IFN-γ or IFN-γR prevents renal disease in these mice (Ozmen et al., 1995). With respect to the nervous system, it has been shown that loss of IFN-γR can reduce the autoimmune phenotype of experimental autoimmune neuritis, an animal model of the human Guillain–Barré syndrome, an autoimmune disorder of the peripheral nervous system (Fujioka, 2018; Zhu et al., 1994; Hartung et al., 1990). Experimental autoimmune neuritis has been predominantly investigated in rats and can be induced with peripheral myelin antigens in combination with adjuvants, which causes CD4 T cell–mediated demyelination in the peripheral nervous system (Zhu et al., 2001). The response we observe after sciatic denervation and the associated cytokine profile resembles this disease model (Zhang et al., 2013). There is furthermore evidence of damage to the SNS in patients with Guillain–Barré syndrome (Matsuyama and Haymaker, 1967). Thus, expression of IFN-γ, as induced by lack of sympathetic tone, is a critical factor in autoimmunity to peripheral nerves. While the mechanism of the role of IFN-γ in autoimmunity is not entirely clear, in lupus, LN IFN-γ expression has been linked to the generation of T follicular helper cells cells, GC formation, and the production of autoantibodies and hypergammaglobulinemia (Lee et al., 2012). This succession of events matches our observations of enhanced GC B cell formation and autoantibody generation. Together, our data provide a mechanistic link between loss of direct sympathetic input to the LN and autoimmunity to peripheral nerves.

We have identified lack of local innervation, as well as loss of sympathetic tone, to cause an increase in IFN-γ expression in CD8 T cells and demonstrated that this increase was not dependent on signals the LN received via blood or lymph, indicating that resident CD8 T cells were responsible for the observed effect and that the nerve signal acts directly on the LN. Furthermore, we have shown LN CD8 T cells to express the Adrb2 and to down-regulate IFN-γ expression after clenbuterol treatment in a reductionist in vitro setting, indicating that they can be direct targets of adrenergic nerves. In the LN, sympathetic nerve fibers enter the LN alongside arteries, but branching of nerves into the LN parenchyma has been observed. Indeed, we also detected some sympathetic nerve fibers in the T cell zone, potentially allowing direct nerve–immune cell interactions. Von Andrian and colleagues (Huang et al., 2021) recently mapped the cells that are directly innervated by sensory nerves in the LN, identifying them to be predominantly stromal cells, including blood and lymphatic endothelial cells. However, we hardly detected IFN-γ expression in stromal cells (data not shown), indicating that innervation of sympathetic nerves and/or the reaction to sympathetic input may differ from sensory nerves. In addition, the response of the LN to loss of neural input in any form will be markedly different from a response caused by the stimulation of nerves. Clearly, these data warrant the need for future investigations to functionally define the specific transmission mechanisms of sympathetic nerve signaling to the LN.

In addition to IFN-γ–producing CD8 T cells, our data implicate signals reaching the LN via afferent lymph in this process. This signal is likely the antigen draining to the denervated LN. While we did not detect a potential role for DCs in antigen processing and the overall response, it is possible that other phagocyte subsets in the LN such as subscapular sinus macrophages are involved, which can filter afferent lymph. The interplay between IFN-γ–producing CD8 T cells and antigen–presenting cells within the popLN in response to nerve injury, as well as potential additional intermediate cells that express the Adrb2 and may relay nerve signals via distinct chemokines and cytokines (van de Pavert et al., 2009) to their surroundings, will need to be investigated in future studies. Together, our data demonstrate that sciatic nerve injury and loss of sympathetic tone directly cause an increase in IFN-γ production in LN CD8 T cells, causing LN expansion.

## Material and methods

### Mice

8–12-wk-old male WT C57BL6/NCrl or C57BL/6JRj mice were purchased from Charles River or Janvier Labs for denervation experiments. 9–20-wk-old *Tcr^OTII^* mice were kindly provided by Susanne Stutte (Biomedical Center, Ludwig-Maximillians-Universität München). The other genetically modified murine strains, including *Myd88^−/−^* (The Jackson Laboratory), *Clec9aCre*, *Rosa-iDTR*, CGRP-KO (Mutant Mouse Resource and Research Center), and *Adrb2^−/−^* (a gift from Gérard Karsenty [Columbia University Irving Medical Center, New York, NY] through Paul Frenette [The Ruth L. and David S. Gottesman Institute for Stem Cell and Regenerative Medicine Research, Albert Einstein Colle of Medicine, New York, NY]) mice were used as males between 8 and 12 wk of age. Mice were housed under a 12 h light:12 h dark cycle with unlimited access to food and water. Cervical dislocation under isoflurane was applied at indicated end points for animal euthanization. All animal procedures and experiments were in accordance with the ministry of animal welfare of the region of Oberbayern and with the German law of animal welfare or were approved and performed in accordance with the guidelines of the animal research committee of Geneva.

### Reagents

Information of commercial kits, reagents, chemicals, antibodies, and primers are provided as supplemental material (Table S1, Table S2, Table S3, Table S4, Table S5, and Table S6).

### Surgery

Mice were anesthetized by i.p. injection of ketamine (100 mg/kg; Medistar) and xylazine (20 mg/kg; Rompun; Bayer Vital GmbH). Prior to surgical procedures, mice were shaved at indicated sites using a veterinarian clipper (Aesculap; GT415; Braun) and depilation cream (Veet). Hair removal and incision sites of each surgery are described individually below. Surgery was performed on a heated pad to reduce heat loss under anesthesia, and the incision area was thoroughly cleaned with ethanol. After surgical procedures, surgical wounds were sutured with sterile vicryl-coated 6–0 suture (V991H Ethicon; Johnson & Johnson Medical Ltd). From the day of surgery onwards, mice received buprenorphine (0.1 mg/kg; Temgesic; Indivior UK Limited) s.c. twice a day for 4 d consecutively.

For sciatic denervation and transplantation, hairs on the dorsal (lateral) side of the thighs were shaved along the line of the femur. After opening the skin on the dorsal side along the femur, connective tissues between the biceps femoris muscle and the quadriceps femoris muscle in the thigh were carefully teased apart to expose the sciatic nerve. The sciatic nerve was then cut twice, 3 mm apart, and the section between incisions was removed to leave a clear gap at hip level. For autologous sciatic transplantation, the excised piece of the sciatic nerve was transplanted into the space between the biceps femoris and quadriceps femoris of the contralateral leg (receiving sham surgery). To assess the effects of sciatic denervation on lymphatic flow, transportation times of intraplantarly injected 1% Evans Blue (E2129; Sigma-Aldrich) solution from the footpad to collecting afferent lymphatics and the popLN were measured, following the surgical opening of the skin area covering the calf and the popliteal fossa under anesthesia.

For femoral denervation, the skin on the ventral side of the thighs was depilated. The femoral nerve was carefully separated from the vessel bundle consisting of the femoral artery and vein, and cut to leave a 3-mm gap in the nerve fiber.

For the denervation at ankle level, the skin surrounding the ankle area was depilated. Incision of the skin was made slightly higher than the joint, and sciatic nerve branches were resected for 2 mm.

For disconnection of afferent lymphatics to the popLN, the skin covering the popliteal fossa was depilated as described (Hendriks, 1978). Briefly, popLNs were carefully disconnected from surrounding tissues, with the exception of the hilum region to leave blood connection and the efferent lymphatic(s) intact. An electrocauter was then used to coagulate the surface of surrounding tissues to prevent them from reconnecting. Afterward, the popLN was pulled slightly out and secured by tying to surrounding connective tissue. To do this, a small loop of fine suturing thread (T04A10Q07-13; AROSurgical) was made to lasso the popLN from the remaining blood vessel connections. On the day of popLN analysis, 1% Evans Blue (E2129; Sigma-Aldrich) was injected intraplantarly to mice under anesthesia to verify the efficacy of lymphatic disconnection of the popLN. PopLNs still receiving lymph-borne dye were excluded from experiments.

### Local phenol-mediated neurolysis

We optimized a previously described phenol-induced neurolysis method (Sung et al., 2001) in this study. Briefly, the sciatic nerve was surgically exposed at hip level, and 3 mm of the sciatic nerve was enwrapped with 5% phenol–soaked cotton swabs for 20 min. Plastic cylinders made of truncated 200 µl Pipetman tips were used to encapsulate the wrapping unit to protect the surrounding tissues from coming in contact with phenol. The wound was closed with a suture, and the popLN was analyzed a week afterward.

### Surface blood perfusion measurement

Surface blood perfusion measurements of the murine hindlimbs were performed using the laser Doppler imaging technique (Moor LDI 5061 and Moor Software Version 3.01; Moor Instruments) as previously described (Lasch et al., 2019). Measurements were performed 1 wk and 2 wk after unilateral sciatic denervation.

### Flow cytometry

Blood samples were collected and subjected to RBC lysis (using RBC lysis buffer: 0.154 M NH_4_Cl, 0.05 mM EDTA, and 10 mM KHCO_3_, pH 7.25). LN samples were minced with scissors in calcium and magnesium containing Dulbecco’s PBS (DPBS) with DNase I (200 µg/ml) and collagenase IV (1 mg/ml). Skin samples were minced with scissors in DPBS with DNase I (200 µg/ml), collagenase IV (1 mg/ml), and dispase II (2 U/ml). Sciatic nerve samples were minced with scissors in DPBS with dispase II (2 U/ml) and collagense D (0.5 mg/ml). Digestion tubes were slowly rotated and incubated at 37°C for 30 min. Afterward, the digested sample was gently ground against a 70-micron cell strainer and rinsed with cold PEB (2 mM EDTA and 2% FBS in PBS).

Single-cell suspensions were incubated with Fc-receptor blocking antibodies (anti-CD16/32) on ice for 20 min, followed by incubation in fluorescence-conjugated antibody mix on ice for another 30 min. Samples were washed with PEB and then taken up in PEB containing 0.3 µM DAPI.

For intracellular staining of Foxp3, single-cell suspensions were stained with Zombie violet viability dye (Biolegend) followed by the other fluorescence-conjugated antibodies except anti-mouse Foxp3–Alexa 647. Stained samples were fixed and permeabilized using the True-Nuclear transcription factor buffer set (Biolegend) before incubation with anti-mouse Foxp3–Alexa 647 on ice for 30 min.

For intracellular staining of IFN-γ, 300,000 cells were cultured in cell culture medium (RPMI-1640 with FBS, L-glutamate, and penicillin/streptomycin) with phorbol 12-myristate 13-acetate (10 ng/ml) and ionomycin (1 µg/ml) on a 96-well U-bottom plate for 2 h, followed by adding brefeldin A (1 µg/ml) for another 3 h culture (in some experiments, in the presence or absence of clenbuterol). Cells were cultured at 37°C with 5% carbon dioxide. Afterward, cells were collected and incubated with a master mix containing Fixable Viability Dye 780 (eBioscience) and desired staining antibodies for surface markers. Stained cells were then fixed and permeabilized using the Intracellular Fixation & Permeabilization Buffer Set (eBioscience) followed by cytoplasmic staining of IFN-γ using anti-mouse IFN-γ–PE-Dazzle 594.

### Local skin disinfection

After surgery, mouse legs and footpads were immersed in 75% ethanol or 0.005% hypochlorite for 10 s for transient removal of surface microbes.

### Adoptive transfer assays

Splenic and LN single-cell suspensions were obtained by gently grinding tissues against 70-micron cell strainers without digestion. Splenic and LN cells were mixed at a 50:50 ratio and incubated with 1.5 µM CFSE in PBS at 37°C for 20 min. Afterward, labeled cells were washed three times with 37°C PEB. On D7 after surgery, each mouse received 2 × 10^7^ donor cells via i.v. injection. Remaining donor cells were subjected to flow cytometry to examine the labeling efficacy and donor cell composition. 2 h after labeled donor cells were transferred to unilaterally denervated mice, their popLNs were harvested and processed. Cells were analyzed by flow cytometry.

### Homing blockade

Blocking leukocyte homing to LNs was conducted by i.p. injection of blocking antibodies at D1 and D4. Antibodies were diluted in PBS to the working concentration. Blocking was achieved by injection of either combined anti-integrin α4/αL (100 µg each antibody/mouse) antibodies or their isotype control antibodies (100 µg each antibody/mouse) or injection of anti–L-selectin antibody (200 µg/mouse) or its isotype control antibody (200 µg/mouse).

### Retention assays

CFSE-labeled cells were adoptively transferred into mice. 2 h later, LN entry was blocked with antibodies (see Homing blockade). Mice were sacrificed 0 h and 18 h after injection of blocking antibodies, allowing for a calculation of the CFSE-labeled remaining fraction 18 h after homing blockade.

### MHCII blockade

Either anti-MHCII antibody (500 µg/mouse) or its isotype control antibody (500 µg/mouse) was injected i.p. at D0 and D4. The antibody was diluted in PBS to the working concentration.

### Cytokine blockade

Antibodies against mouse TNF-α, IL-1α, IL-1β, and/or IL-6 were injected i.p. individually or in combination to mice at D0 (3 h before operation) and D1 in a dose of 100 µg per mouse to neutralize cytokines. The control group received respective isotype antibodies. Anti–IFN-γ (200 µg/mouse) was given in the same way but at D0 and D3.

### Cell type–specific depletion assays

To deplete neutrophils, each mouse was injected i.p. with 200 µg of either isotype control antibody (LTF-2) or anti-Ly6G antibody (1A8) at D0 and D4. To eliminate NK cells, each mouse was injected i.p. with 200 µg of either isotype control antibody (C1.18.4; BE0085; BioXCell) or anti-NK1.1 antibody (PK136; BE0036; BioXCell) at D1 and D4. When organs were harvested at D7, spleen and blood were taken to verify the depletion efficacy. To deplete CD8 cells, each mouse was injected i.p. with 200 µg of either isotype control antibody (LTF-2) or anti-CD8 antibody (2.43) at D1, D2, and D5. Depletion efficacy was assessed with another clone of anti-mouse CD8 antibody (53–6.7).

To deplete DCs, Clec9a-DTR (diphtheria toxin receptor) or inducible diphtheria toxin receptor (iDTR) mice were injected i.p. with diphtheria toxin at the surgery day (D0) 4 h before operation and the day after (D1). popLNs were harvested at D2 to quantify immune subsets.

### qPCR

LN samples were immersed in 200 µl QIAzol lysis reagent (79306; Qiagen) in innuSPEED Lysis Tubes (845-CS-1020050; AJ Innuscreen GmbH) and homogenized using SpeedMill PLUS (Analytik Jena AG). RNA was separated from DNA and protein by mixing the QIAzol lysate with 40 µl chloroform (C2432; Sigma-Aldrich). RNA from the aqueous layer was then precipitated in isopropanol (A3928; Reac AppliChem ITW Reagents), washed with 75% ethanol, and dissolved in water. Potential residual DNA in the extract was digested by recombinant DNase I, and the remaining RNA was further purified using the RNeasy MiniElute Cleanup Kit (Qiagen). Cell type–specific RNA was prepared by cell sorting followed by spin column–based RNA extraction using the RNeasy Plus Micro kit (Qiagen).

Conversion of RNA to cDNA was performed using the PrimeScript RT Reagent Kit (RR037A; Takara Bio). Samples were then diluted in water to make a 1 ng RNA/µl working cDNA solution. cDNA samples were stored at −20°C, and RNA samples at −80°C.

Genes were quantified by qPCR using cDNA and Fast SYBR Green Master Mix (4385614; Applied Biosystems). The reaction solution contained 1 µl working cDNA solution from each sample, primers of target genes, water, and Fast SYBR Green Master Mix. The qPCR program was 95°C for 10 min, 40 cycles of 95°C for 15 s, and 62°C for 1 min. The melting curve was measured using the machine’s built-in setting.

### Serum isotyping

For isotyping, sera were diluted (1:10,000) and used on the Pierce Rapid ELISA Mouse mAb Isotyping Kit (37503; Invitrogen). Briefly, 50 µl samples were mixed with 50 µl of anti-mouse antibodies–HRP solution and transferred to precoated flat-bottom wells. After 1 h incubation, the sample-antibody solution was discarded and washed off with Tris-buffered saline with 0.05% Tween 20. 75 µl 3,3′,5,5′-tetramethylbenzidine substrate solution was added at room temperature for 5 min, followed by adding 75 µl stop solution. Absorbance of 450 and 570 (reference wavelength) nm was measured by a TECAN plate reader (SPARK 10M; TECAN).

### Total IgG quantification

For quantification of total circulating IgG, Mouse IgG Total Ready-SET-Go! (88–50400-86; Invitrogen) was used with diluted sera (1:10,000 or 1:20,000). Briefly, flat-bottom 96-well plates were coated with capture antibody by adding diluted capture antibody (250×) to the plates and incubating at 4°C overnight. The next day, the coating solution was discarded, and the plates were washed with PBS with 0.05% Tween 20. 250 µl blocking solution was added to the plates and left for 2 h at room temperature. The blocking solution was discarded, and plates were washed with PBS with 0.05% Tween 20. Prior to applying standards and samples, the standards were prepared based on twofold serial dilution. 200 µl standards or samples as well as 50 µl of detection antibody were added to the plate and incubated at room temperature for 3 h. Samples were washed using PBS with 0.05% Tween 20. 3,3′,5,5′-tetramethylbenzidine substrate solution was added at room temperature for 15 min, followed by adding 100 µl stop solution. Absorbance of 450 and 570 (reference wavelength) nm was measured by a TECAN plate reader (SPARK 10M; TECAN).

### Detection of anti-nuclear antibodies

Sera were diluted to 10 µg/ml and applied to HEp-2 slides (ORG870; Orgentec) for anti-nuclear antibodies. Samples of 25 µl were added to precoated slides in a moisture chamber at 4°C for 30 min. After the incubation, the slides were rinsed and immersed in PBS for 5 min. After drying of excessive PBS, detection antibodies were applied to the slides and incubated for 30 min. Slides were carefully washed with PBS and dried off, followed by being covered with coverslips. Images were acquired on a Zeiss Examiner D1 spinning-disk confocal microscope using SlideBook 6 software (3i; Intelligent Imaging Innovations), and the images were processed with Fiji.

### Immunofluorescence

PopLN samples were subjected to whole-mount or section staining, and the sciatic nerve was processed as cross-sectioned slices. To prepare on-slide staining of tissue sections, samples of the sciatic nerve, the popLN, or the muscle surrounding the popLN were harvested and placed in Cryomolds (4557; Tissue-Tek; Sakura) with OCT compound (4583; Tissue-Tek; Sakura) followed by snap-freezing on dry ice. The embedded samples were cut on a Leica CM3050 S cryostat. All steps in the staining procedures were performed at room temperature except primary antibody staining, which was performed at 4°C.

For whole-mount imaging of sympathetic nerves in the popLN, the popLN was harvested and cut in half using a scalpel blade. Samples were fixed in 4% paraformaldehyde (PFA) for 30 min. After fixation, samples were immersed in the blocking cocktail containing 20% goat serum (31872; Invitrogen), 0.5% Triton X-100 (M143; Amresco) and 20% (vol/vol) streptavidin solution (SP-2002; Vector Laboratories) in PBS for 2 h. For primary antibody staining, anti-TH (1:1,000) and anti-CD31–Alexa 647 (1:100) antibodies, 2% goat serum, and 20% (vol/vol) biotin solution (SP-2002; Vector Laboratories) in PBS were applied on samples for overnight staining. The following day, the primary antibody solution was washed off with PBS and replaced with goat anti-rabbit-biotin secondary antibody (1:500) in PBS with 2% goat serum. Samples were stained with this secondary antibody solution for 2 h and washed off with PBS before incubation with streptavidin-Cy3 (1:500) in PBS for 30 min. All the staining procedures were performed in the dark.

To identify functional processes and cell types in the popLN, 10-µm sections were made. Samples were fixed with 4% PFA for 10 min and then blocked with 20% goat serum and 0.5% Triton X-100 in PBS for 30 min. Sera (IgG 1.25 µg/µl) or fluorescence-conjugated primary antibodies recognizing mouse CD4 (1:100), CD8 (1:100), B220 (1:100), Ki67 (1:100), and GL7 (1:100) were used in PBS containing 2% goat serum to stain samples overnight. For serum staining, anti-mouse CD16/32 (1:100) was supplemented in the blocking master mix, and FITC-labeled secondary antibody against mouse IgG (1:1,000) was incubated with samples, before and after application of primary antibody, respectively.

For imaging the sciatic nerve, 20-µm cross-section slices were made. Samples were fixed with 4% PFA for 10 min and then blocked with 20% goat serum, 0.5% Triton X-100, and 20% (vol/vol) streptavidin solution in PBS for 2 h. Sera (IgG 1.25 µg/µl) or primary antibodies reacting to CD31 (1:100), MRP-14 (1:100), JAM-C (1:100), MPZ (1:100), NF-H (1:100), NF-M (1:100), and TH (1:1,000) were used in PBS containing 2% goat serum and 20% (vol/vol) biotin solution to stain overnight. The following day, primary antibody solution was washed off, and secondary antibodies (goat anti-mouse IgG-FITC, biotinylated goat anti-rabbit, and/or fluorescence-conjugated goat anti-chicken; 1:1,000) in PBS with 2% goat serum were applied for 2 h and washed off with PBS before, if needed, incubation with streptavidin-Cy3 (1:500) in PBS for 30 min.

Images were acquired on a Zeiss Examiner D1 spinning-disk confocal microscope using SlideBook 6 software (3i; Intelligent Imaging Innovations). Scans of LN sections and whole mounts were taken with 100× magnification (10× ocular and 10× objective). Cross-sections of sciatic nerves were imaged with 630× magnification (10× ocular and 63× objective). All images were reconstructed using auto-alignment tools of SlideBook 6. Rebuilt volumetric scans were flattened by maximum projection. Brightness, contrast, colors, and overlay of images were adjusted using Fiji software (ImageJ). For investigations of protein expression in HEVs, all quantifications were performed using mask analysis (Zeiss software) based on PECAM-1 expression and quantifying expression of other fluorescent channels within the mask containing PECAM^+^ pixels, delineating HEV structures.

### Chemical sympathectomy

To ablate sympathetic tones in the periphery, we used a chemical method using 6-OHDA as previously described (Lucas et al., 2013; Scheiermann et al., 2012). Mice were treated i.p. with 100 mg/kg 6-OHDA (with ascorbate 20 mg/ml in saline) on D0, and 250 mg/kg on D2. Chemically sympathectomized mice were analyzed from D5.

### Treatment with nerve-derived substances

CGRP (1 µg/mouse; 1161; Tocris) and substance P (250 µg/kg; 1156; Tocris) were dissolved in PBS and injected i.p. to mice, separately or combined. The Adrb2 selective agonist clenbuterol (5 mg/kg) was i.p. injected to mice daily.

### Statistical analysis

Fluorescence intensity of target molecules from image data were quantified with Fiji software using original files without thresholding. The masks of regions of interest were created with costained markers with minimal thresholding to define target areas. Cell counts were quantified by total cell count of samples and subset fractions, which were determined by a Coulter counter (Z2 analyzer; Beckman Coulter) and flow cytometry.

Data from the same experimental setting were pooled and analyzed as a collective dataset. Data were analyzed using Prism 7 (GraphPad). Outliers of each dataset were detected and eliminated using a built-in function of outlier detection in Prism 7 with false discovery rate of 1%. After clearance of outliers, data were presented as mean ± SEM.

For data comparison between control and experimental group(s), to compare effects between two groups with one variable, the unpaired Student’s *t* test was used. To compare effects across multiple groups (more than two) with one variable, one-way ANOVA and optionally Tukey’s post-test were used for further comparison between each pair. Two-way ANOVA was used to analyze the effects of multiple independent variables on outcomes of the surgery. Šídák’s post-test was used for comparison between two groups within a variable. The Wilcoxon test was performed for paired analyses of non-Gaussian–distributed data.

All data are presented as mean ± SEM: *, P < 0.05; **, P < 0.01; ***, P < 0.001; and ****, P < 0.0001.

### Online supplemental material

Fig. S1 shows the general gating strategy for leukocytes and stromal cells as well as the influence of surgeries on the local circulation and muscle inflammation. Fig. S2 provides information about migration routes of immune subsets to the LN after denervation. Fig. S3 characterizes the B cell response after denervation. Fig. S4 describes the autoimmune phenotype after denervation. Fig. S5 shows the role of different leukocyte subsets in the reaction. Table S1 lists the depletion and neutralization antibodies. Table S2 lists the immunofluorescence antibodies. Table S3 lists flow cytometry antibodies. Table S4 shows the chemicals and biological compounds. Table S5 lists the commercial kits. Table S6 lists the forward and reverse primers.

## Supplementary Material

Table S1 lists the depletion and neutralization antibodies.

Table S2 lists the immunofluorescence antibodies.

Table S3 lists flow cytometry antibodies.

Table S4 shows the chemicals and biological compounds.

Table S5 lists the commercial kits.

Table S6 lists the forward and reverse primers.
