# Supplementary material for: Loss of direct adrenergic innervation after peripheral nerve injury causes lymph node expansion through IFN-γ
Source: J Exp Med. 2021 Jun 4;218(8):e20202377. doi: 10.1084/jem.20202377 (PMC8185988; doi:10.1084/jem.20202377)
Supplement: Table S1 — lists the depletion and neutralization antibodies. [file JEM_20202377_TableS1.docx]

Table S1. Depletion and neutralization antibodies

| Anti-mouse Ly6G (1A8) | BioXCell | BE0075 |
| --- | --- | --- |
| Anti-mouse NK1.1 (PK136) | BioXCell | BE0036 |
| Anti-mouse α_4_-integrin/VLA-4 (PS/2) | BioXCell | BE0071 |
| Anti-mouse α_L_-integrin/LFA-1α (M17/4) | BioXCell | BE0006 |
| Anti-mouse L-selectin (MEL-14) | BioXCell | BE0021 |
| Anti-mouse MHCII (Y3P) | BioXCell | BE0178 |
| Anti-mouse IFN-γ (XMG1.2) | BioXCell | BE0055 |
| Anti-mouse TNF-α (XT3.11) | BioXCell | BE0058 |
| Anti-mouse IL-1α (ALF161) | BioXCell | BE0243 |
| Anti-mouse IL-1β (B122) | BioXCell | BE0246 |
| Anti-mouse IL-6 (MP5-20F3) | BioXCell | BE0046 |
| Anti-mouse CD8 (2.43) | BioXCell | BE0061 |
| Rat IgG1 isotype control, anti-HRP (HRPN) | BioXCell | BE0088 |
| Armenian hamster IgG control (polyclonal) | BioXCell | BE0091 |
| Rat IgG2a isotype control, anti- Trinitrophenol (2A3) | BioXCell | BE0089 |
| Rat IgG2b isotype control, anti-KLH (LTF-2) | BioXCell | BE0090 |
| Mouse IgG2a isotype control, unknown specificity (C1.18.4) | BioXCell | BE0085 |
