# Supplementary material for: Loss of direct adrenergic innervation after peripheral nerve injury causes lymph node expansion through IFN-γ
Source: J Exp Med. 2021 Jun 4;218(8):e20202377. doi: 10.1084/jem.20202377 (PMC8185988; doi:10.1084/jem.20202377)
Supplement: Table S2 — lists the immunofluorescence antibodies. [file JEM_20202377_TableS2.docx]

Table S2. Immunofluorescence antibodies

| Rabbit anti-mouse tyrosine hydroxylase (polyclonal) | Merck Millipore (Chemicon) | AB152 |
| --- | --- | --- |
| Chicken anti-mouse myelin P0 (polyclonal) | NOVUS Biologicals | NB100-1607 |
| Chicken anti-mouse neurofilament H (polyclonal) | Merck Millipore (Chemicon) | AB5735 |
| Chicken anti-mouse neurofilament M (polyclonal) | Merck Millipore (Chemicon) | AB5539 |
| Rabbit anti-mouse neurofilament H (polyclonal) | Synaptic Systems | 171102 |
| Rabbit anti-mouse neurofilament M (polyclonal) | Synaptic Systems | 171202 |
| Biotinylated goat anti-rabbit IgG (VECTASTAIN ABC kit) | Vector Laboratories | PK-4001 |
| Goat anti-chicken IgY-DyLight 405 (polyclonal) | Jackson Lab | AB_2337389 |
| Goat anti-chicken IgY-Alexa 488 (polyclonal) | Thermo Fisher Scientific | A11039 |
| Goat-anti rabbit IgG-Alexa 488 (polyclonal) | Thermo Fisher Scientific | A11034 |
| Goat-anti rabbit IgG-Alexa 568 (polyclonal) | Thermo Fisher Scientific | A11011 |
| Goat-anti rabbit IgG-Alexa 633 (polyclonal) | Thermo Fisher Scientific | A21070 |
| Anti-mouse Ki67-PE (SolA15) | eBioscience | 53-6036-80 |
| Goat anti-mouse IgG (polyclonal) | Biolegend | 405301 |
| Goat anti-mouse IgG-FITC (polyclonal) | Biolegend | 405305 |
| Rabbit anti-mouse MRP-14 (polyclonal) | gift from Dr. Monika Prünster, LMU Munich | N/A |
| Anti-mouse JAM-C-APC (209628) | R&D Systems | FAB7050A |
| Anti-mouse CD31-Alexa 647 (MEC13.3) | Biolegend | 102516 |
| Rabbit anti-mouse tyrosine hydroxylase (polyclonal) | Merck Millipore | AB152 |
| Anti-mouse ICAM-1-PE (YN1/1.7.4) | Biolegend | 116108 |
| Anti-mouse VCAM-1-PE (429) | Biolegend | 105714 |
| Anti-mouse CD4-APC (GK1.5) | Biolegend | 100412 |
| Anti-mouse CD8-Alexa 647 (53-6.7) | Biolegend | 100724 |
| Anti-mouse B220-Alexa 488 (RA3-6B2) | Biolegend | 103225 |
| Anti-mouse GL7-Alexa 647 (GL7) | Biolegend | 144606 |
| Anti-mouse CD16/32 (93) | Biolegend | 101302 |
