# Supplementary material for: Loss of direct adrenergic innervation after peripheral nerve injury causes lymph node expansion through IFN-γ
Source: J Exp Med. 2021 Jun 4;218(8):e20202377. doi: 10.1084/jem.20202377 (PMC8185988; doi:10.1084/jem.20202377)
Supplement: Table S3 — lists flow cytometry antibodies. [file JEM_20202377_TableS3.docx]

Table S3. Flow cytometry antibodies

| Anti-mouse GL7-Alexa 647 (GL7) | Biolegend | 144606 |
| --- | --- | --- |
| Anti-mouse CD16/32 (93) | Biolegend | 101302 |
| Anti-mouse CD45-FITC (30-F11) | Biolegend | 103107 |
| Anti-mouse CD45-BUV 395 (30-F11) | BD Bioscience | 565967 |
| Anti-mouse CD31-APC (MEC13.3) | Biolegend | 102509 |
| Anti-mouse CD31-PerCP/Cy5.5 (390) | Biolegend | 102419 |
| Anti-mouse podoplanin-PE (8.1.1.) | Biolegend | 127407 |
| Anti-mouse CD3-PE/Dazzle 594 (17A2) | Biolegend | 100246 |
| Anti-mouse CD3-BV 711 (17A2) | Biolegend | 100241 |
| Anti-mouse NK1.1-Alexa 700 (PK136) | eBioscience | 55-5941-82 |
| Anti-mouse CD4-BV 570 (RM4-5) | Biolegend | 100542 |
| Anti-mouse CD4-BV 605 (RM4-5) | Biolegend | 100548 |
| Anti-mouse CD4-Alexa 488 (RM4-5) | Biolegend | 100532 |
| Anti-mouse CD8-PE (53-6.7) | Biolegend | 100707 |
| Anti-mouse CD8-BV 650 (53-6.7) | Biolegend | 100741 |
| Anti-mouse CD169-Alexa 647 (3D6.112) | Biolegend | 142408 |
| Anti-mouse CD11c-APC/Cy7 (N418) | Biolegend | 117323 |
| Anti-mouse CD11c-PerCP/Cy5.5 (N418) | Biolegend | 117327 |
| Anti-mouse CD11c-BV 510 (N418) | Biolegend | 117337 |
| Anti-mouse B220-PE/Cy7 (RA3-6B2) | Biolegend | 103222 |
| Anti-mouse B220-PE (RA3-6B2) | Biolegend | 103207 |
| Anti-mouse B220-BV 421 (RA3-6B2) | Biolegend | 103239 |
| Anti-mouse Gr1-PerCP/Cy5.5 (RB6-8C5) | Biolegend | 108428 |
| Anti-mouse Gr1-FITC (RB6-8C5) | Biolegend | 108406 |
| Anti-mouse MHCII-PE/Cy7 (M5/114.15.2) | Biolegend | 107629 |
| Anti-mouse MHCII-Alexa 647 (M5/114.15.2) | Biolegend | 107617 |
| Anti-mouse MHCII-Alexa 700 (M5/114.15.2) | Biolegend | 107621 |
| Anti-mouse CD11b-BV 785 (M1/70) | Biolegend | 101243 |
| Anti-mouse CD11b-BUV 737 (M1/70) | BD Bioscience | 612801 |
| Anti-mouse F4/80-PE (BM8) | Biolegend | 123110 |
| Anti-mouse IFN-γ-PE/Dazzle 594 (XMG1.2) | Biolegend | 505845 |
| Anti-mouse CD25-Alexa 488 (PC61) | Biolegend | 102017 |
| Anti-mouse Foxp3-Alexa 647 (150D) | Biolegend | 320014 |
| Anti-mouse CD138-APC/Cy7 (281-2) | Biolegend | 142530 |
| Anti-mouse Fas-PE/CF 594 (Jo2) | BD Bioscience | 562499 |
| Anti-mouse CD49b-PE/Cy7 (DX5) | Biolegend | 108922 |
| Anti-mouse CD24-BV605 (M1/69) | Biolegend | 101827 |
| Anti-mouse CD172a-PE/Cy7 (P84) | Biolegend | 144007 |
| Anti-mouse F4/80-Alexa 647 (BM8) | Biolegend | 123121 |
| Anti-mouse XCR1-BV 421 (ZET) | Biolegend | 148216 |
| Anti-mouse β2-Adrenergic Receptor-FITC | Alomone Labs | AAR-016-F |
| Rabbit IgG Isotype Control-FITC | eBioscience | 11-4614-80 |
