# Supplementary material for: Loss of direct adrenergic innervation after peripheral nerve injury causes lymph node expansion through IFN-γ
Source: J Exp Med. 2021 Jun 4;218(8):e20202377. doi: 10.1084/jem.20202377 (PMC8185988; doi:10.1084/jem.20202377)
Supplement: Table S4 — shows the chemicals and biological compounds. [file JEM_20202377_TableS4.docx]

Table S4. Chemicals and biological compounds

| CFSE | Thermo Fisher Scientific | C34554 |
| --- | --- | --- |
| Evans Blue | Sigma | E2129 |
| DNase I | Reac AppliChem ITW Reagents | A3778 |
| Collagenase IV | Sigma | C5138 |
| Diphtheria Toxin | Sigma | D0564 |
| Phenol solution | Sigma | P4557 |
| Control liposome | Liposoma BV | P-005 |
| Clodronate liposome | Liposoma BV | C-005 |
| 6-hydroxydopamine (6-OHDA) | Sigma | 162957 |
| Streptavidin-BV 421 | Biolegend | 405226 |
| Streptavidin-DyLight 488 | Biolegend | 405218 |
| Streptavidin-Cy3 | Biolegend | 405215 |
| Streptavidin-APC | Biolegend | 205207 |
| Biotin/Streptavidin blocking kit | Vector Laboratories | SP-2002 |
| 4',6-diamidino-2-phenylindole (DAPI) | Biolegend | 422801 |
| QIAzol | Qiagen Hilden Germany | 79306 |
| Ethanol | Reac AppliChem ITW Reagents | A3678 |
| Isopropanol | Reac AppliChem ITW Reagents | A3928 |
| Chloroform | Sigma | C2432 |
| Normal goat serum | Invitrogen | 31872 |
| Fetal bovine serum | Gibco | 1500-064 |
| 0.5 M EDTA pH 8.0 | Thermo Fisher Scientific | AM9261 |
| Dulbecco’s PBS | Gibco | 14040-083 |
| NH_4_Cl | Merck KGaA | EMSURE 1.04854.0500 |
| KHCO_3_ | Merck KGaA | EMSURE 1.01145.0500 |
| Triton X100 | Amresco | M143 |
| Tween 20 | Sigma | P9416 |
| 4% paraformaldehyde solution | Affymetrix | 12777847 |
| Collagenase D | Roche | 11088858001 |
| Fixable Viability Dye eFluor™ 780 | eBioscience | 65-0865-14 |
| Brefeldin A Solution (1000x) | Biolegend | 420601 |
| phorbol 12-myristate 13-acetate | Sigma | P8139 |
| Ionomycin | Sigma | I9657 |
| calcitonin gene-related peptide | Tocris | 1161 |
| substance P | Tocris | 1156 |
| Clenbuterol | Sigma | C5423 |
| Dispase II | Gibco | 17105041 |
