# Supplementary material for: Loss of direct adrenergic innervation after peripheral nerve injury causes lymph node expansion through IFN-γ
Source: J Exp Med. 2021 Jun 4;218(8):e20202377. doi: 10.1084/jem.20202377 (PMC8185988; doi:10.1084/jem.20202377)
Supplement: Table S5 — lists the commercial kits. [file JEM_20202377_TableS5.docx]

Table S5. Commercial kits

| HEp-2 slides | Orgentec | ORG870 |
| --- | --- | --- |
| Pierce Rapid ELISA Mouse mAb Isotyping Kit | Invitrogen | 37503 |
| Mouse IgG total Ready-SET-Go! | Invitrogen | 88-50400-86 |
| RNeasy MinElute Cleanup kit | Qiagen | 74204 |
| RNeasy Plus Mini Kit | Qiagen | 74134 |
| RNeasy Plus Micro Kit | Qiagen | 74034 |
| PrimeScript™ RT Reagent Kit | Takara Bio | RR037A |
| Fast SYBR™ Green Master Mix | Applied Biosystems | 4386514 |
| True-Nuclear Transcription Factor Buffer Set | Biolegend | 424401 |
| Intracellular Fixation & Permeabilization Buffer Set | eBioscience | 88-8824-00 |
| Zombie Violet fixable viability kit | Biolegend | 423113 |
| Fixable viability dye eFluor 780 | Invitrogen | 650865 |
