# Supplementary material for: Loss of direct adrenergic innervation after peripheral nerve injury causes lymph node expansion through IFN-γ
Source: J Exp Med. 2021 Jun 4;218(8):e20202377. doi: 10.1084/jem.20202377 (PMC8185988; doi:10.1084/jem.20202377)
Supplement: Table S6 — lists the forward and reverse primers. [file JEM_20202377_TableS6.docx]

Table S6. Primers

| *Rpl32 Forward* | 5′-ACAATGTCAAGGAGCTGGAG-3′ |
| --- | --- |
| *Rpl32 Reverse* | 5′-TTGGGATTGGTGACTCTGATG-3′ |
| *Ccl19 Forward* | 5′-ATGTGAATCACTCTGGCCCAGGAA-3′ |
| *Ccl19 Reverse* | 5′-AAGCGGCTTTATTGGAAGCTCTGC-3′ |
| *Ccl21 Forward* | 5′-TGAACAGACACAGCCCTCAAGA-3′ |
| *Ccl21 Reverse* | 5′-CCTCTTTGCCTGTGAGTTGGA-3′ |
| *Cxcl12 Forward* | 5′-CAGAGCCAACGTCAAGCA-3′ |
| *Cxcl12 Reverse* | 5′-AGGTACTCTTGGATCCAC-3′ |
| *Cxcl13 Forward* | 5′-CATAGATCGGATTCAAGTTACGCC-3′ |
| *Cxcl13 Reverse* | 5′-TCTTGGTCCAGATCACAACTTCA-3′ |
| *Tnfa Forward* | 5′-GCCTCTTCTCATTCCTGCTTG-3′ |
| *Tnfa Reverse* | 5′-CTGATGAGAGGGAGGCCATT-3′ |
| *Tgfb Forward* | 5′-TAAAATCGACATGCCGTCCC-3′ |
| *Tgfb Reverse* | 5′-GAGACATCAAAGCGGACGAT-3′ |
| *Ifng Forward* | 5′-TCAAGTGGCATAGATGTGGAAGAA-3′ |
| *Ifng Reverse* | 5′-TGGCTCTGCAGGATTTTCATG-3′ |
| *Il1a Forward* | 5′-TTGGTTAAATGACCTGCAACA-3′ |
| *Il1a Reverse* | 5′-GAGCGCTCACGAACAGTTG-3′ |
| *Il1b Forward* | 5′-TGTAATGAAAGACGGCACACC-3′ |
| *Il1b Reverse* | 5′-TCTTCTTTGGGTATTGCTTGG-3′ |
| *Il2 Forward* | 5′-AACCTGAAACTCCCCAGGAT-3′ |
| *Il2 Reverse* | 5′-CGCAGAGGTCCAAGTTCATC-3′ |
| *Il4 Forward* | 5′-GGCATTTTGAACGAGGTCACA-3′ |
| *Il4 Reverse* | 5′-GACGTTTGGCACATCCATCTC-3′ |
| *Il6 Forward* | 5′-ACAAGTCGGAGGCTTAATTACACAT-3′ |
| *Il6 Reverse* | 5′-TTGCCATTGCACAACTCTTTTC-3′ |
| *Il7 Forward* | 5′-GTGCCACATTAAAGACAAAGAAG-3′ |
| *Il7 Reverse* | 5′-GTTCATTATTCGGGCAATTACTATC-3′ |
| *Il10 Forward* | 5′-CCCATTCCTCGTCACGATCTC-3′ |
| *Il10 Reverse* | 5′-TCAGACTGGTTTGGGATAGGTTT-3′ |
| *Il12 Forward* | 5′-TACTAGAGAGACTTCTTCCACAACAAGAG-3′ |
| *Il12 Reverse* | 5′-TCTGGTACATCTTCAAGTCCTCATAGA-3′ |
| *Il13 Forward* | 5′-CAGCAGCTTGAGCACATTTC-3′ |
| *Il13 Reverse* | 5′-CGGGATACTGACAGACTCATTT-3′ |
| *Il17a Forward* | 5′-GGACTCTCCACCGCAATGA-3′ |
| *Il17a Reverse* | 5′-GGCACTGAGCTTCCCAGATC-3′ |
| *Il17f Forward* | 5′-CCCCATGGGATTACAACATCAC-3′ |
| *Il17f Reverse* | 5′-CATTGATGCAGCCTGAGTGTCT-3′ |
| Adrb2 Forward | 5′-GTACTGTGCCTAGCCTTAGCGT-3′ |
| *Adrb2 Reverse* | 5′-GGTTAGTGTCCTGTCAAGGAGG-3′ |
